# Gut Microbiome Composition of the Fire Ant Solenopsis invicta: an Integrated Analysis of Host Genotype and Geographical Distribution

**DOI:** 10.1128/spectrum.03585-22

**Published:** 2023-01-05

**Authors:** Qian Xiao, Lei Wang, Si-Qi Chen, Chun-Yan Zheng, Yong-Yue Lu, Yi-Juan Xu

**Affiliations:** a Red Imported Fire Ant Research Center, South China Agricultural University, Guangzhou, China; South China Agricultural University

**Keywords:** invasive ants, symbiotic bacteria, microsatellite, social forms, COI, biogeography

## Abstract

Gut symbiotic bacteria are known to be closely related to insect development, nutrient metabolism, and disease resistance traits, but the most important factors leading to changes in these communities have not been well clarified. To address this, we examined the associations between the gut symbiotic bacteria and the host genotype and geographical distribution of Solenopsis invicta in China, where it is invasive and has spread primarily by human-mediated dispersal. Thirty-two phyla were detected in the gut symbiotic bacteria of *S. invicta*. Proteobacteria were the most dominant group among the gut symbiotic bacteria. Furthermore, the Bray-Curtis dissimilarity matrices of the gut symbiotic bacteria were significantly positively correlated with the geographical distance between the host ant colonies, but this relationship was affected by the social form. The distance between monogyne colonies had a significant effect on the Bray-Curtis dissimilarity matrices of gut symbiotic bacteria, but the distance between polygyne colonies did not. Moreover, the Bray-Curtis dissimilarity matrices were positively correlated with Nei’s genetic distance of the host but were not correlated with the COI-based genetic distance. This study provides a scientific basis for further understanding the ecological adaptability of red imported fire ants during invasion and dispersal.

**IMPORTANCE** We demonstrated that gut microbiota composition and diversity varied among populations. These among-population differences were associated with host genotype and geographical distribution. Our results suggested that population-level differences in *S. invicta* gut microbiota may depend more on environmental factors than on host genotype.

## INTRODUCTION

Gut symbiotic bacteria are closely related to the growth and development of the host. Gut symbiotic bacteria play vital roles in the physiological functions of the host, such as nutrient absorption, immune regulation (1), energy metabolism, and reproductive development (2). For example, symbiotic bacteria involved in the recycling of nitrogen-rich metabolic waste have been found to play an important role in growth and reproduction in *Tetraponera* ants (3). Symbiotic bacteria may help invading ants adapt to the environment and further expand their habitat range (4, 5). Although the importance of symbiotic bacteria has been recognized, the factors that control changes in the gut symbiotic microbiome have not been fully explored, and the interactions among such factors may reveal the evolutionary and ecological relationships between the host and microbiome (6). Gut bacterial symbionts of insects are vertically transmitted from parent to offspring or are environmentally acquired (7). In a study of *Camponotus* ants, Ramalho et al. (8) examined the similarity of microbiota communities based on host collection location and found that environmental acquisition and social transmission may be the main route of transmission of bacterial communities, especially those in the head and thorax.

The environment greatly influences the composition of the symbiotic microbial community and the abundance of its members. High temperature, antibiotics, and insecticides can inhibit the number of yeast-like symbiotic bacteria in brown planthoppers (8, 9). Gut symbiotic bacteria are closely related to food intake and may greatly influence the dietary evolution and species diversity of ants (10). Ant colonies in different habitats and regions may have different gut symbiotic bacteria. Knowles et al. (11) found that environmental factors, especially diet, influence the composition of the host gut microbiome in small mammals (mice, voles, and shrews). Different genotypes of mice fed the same type of diet had consistent intestinal symbiotic bacteria, suggesting that the host diet plays a leading role in shaping gut microbial ecology (12). The significant effect of diet on the intestinal microbiota of the American cockroach Periplaneta americana was also proven, with effects observed on the production of organic acids and CH_4_ by gut bacteria (13). Colonies of the ant genus *Cephalotes*, known as “turtle ants,” fed artificial diets exhibited substantial alteration of their microbial communities (14, 15), and highly similar microbial communities are shared among related and trophically similar ant species (16), suggesting a coevolution between symbiotic bacterial communities and their host. In contrast, diet causes no differences in the bacterial communities of the ant Daceton armigerum (17), indicating that multiple factors consistently structure host-associated microbial communities. However, host genetics seems to be a much more vital determinant than diet. For example, under the same environmental conditions, the relationships among the members of the microbial community reflect the phylogenetic relationship of *Nasonia* wasps, indicating that the structure of the microbial community is closely related to the differentiation of host genes (18). It was proven that host phylogenetic distance accounted for much more of the total variation in beta diversity among *Cephalotes* ant microbiota than did geographic distance (19). Compared with studies on environmental effects, intraspecies studies tend to consider host heredity as having a relatively weak influence, while studies on different species tend to emphasize the role and effect of host heredity (20).

The red imported fire ant, Solenopsis invicta Buren, is one of the most important invasive insects: it can disperse rapidly and is very harmful and aggressive (21, 22). Since their discovery in Taiwan in 2003 and in Guangdong in 2004, these ants have spread widely in southern China (23). A population genetics study could help to reveal the history of the red imported fire ant invasion in China. The colonies of *S. invicta* have two distinct forms: monogyne colonies, each organized around a single queen, and polygyne colonies, containing multiple fertile queens (24). Yang et al. (25) studied the social form and genotypes of different geographic populations of red imported fire ants in China and found that the ratio of polygyne to monogyne colonies was approximately 4:1. A total of three haplotypes were detected, and long-distance jump dispersal was a common method of red imported fire ant spread in China. Huang et al. (26) found that the genetic diversity of red imported fire ants in China is very high, and there is a high degree of genetic differentiation among different populations, which may be caused by geographical isolation and gene flow obstruction. Zhang and Hou (27) used the cytochrome oxidase subunit I (COI) gene to investigate 12 red imported fire ant populations in Fujian Province, and according to their phylogenetic analysis, the populations could be divided into 3 groups belonging to three haplotypes: H22, H5, and H36. It was inferred that Fujian Province may have experienced several red fire ant invasions from other provinces. He et al. (28) analyzed the haplotypes of 13 populations in China based on the COI gene and believed that Hong Kong was the region with the most abundant genetic diversity among the *S. invicta* populations that have invaded China; as all the haplotypes have been reported in the Argentina populations, the researchers speculated that the invasive populations in China came mainly from Argentina or South America. However, another study found that some invasive *S. invicta* populations in China also originate from the United States and possibly other locations (29), suggesting that the red imported fire ant populations in China have a more complex genetic background than previously reported. Previous studies examined the bacterial community composition of *S. invicta* and its colony soils using the pyrosequencing of microbial marker genes (30, 31), and bacteria associated with *S. invicta* colony soils were cultured and isolated (32). However, the composition of the symbiotic bacteria of red imported fire ants with different geographical and genetic backgrounds was not well recognized.

To date, the role of host genetics and the environment in shaping gut microbiota and the relative importance of the two have remained controversial (8, 17, 33–36). The hypothesis that bacteria were acquired from the local environment in which the ant lived was confirmed by feeding (8). The variation of bacterial communities among *D. armigerum* was attributed to the genetic variability of the host (17). Both the host genetics and the environment were considered to be related to gut microbiota. Here, we explore the relationships between the composition of gut symbiotic bacteria of red imported fire ants and the host genotype using highly polymorphic microsatellite markers and COI gene and social form types and their associated microbial communities of geographical latitude pattern in China.

## RESULTS

### Composition and structure of gut symbionts in *S. invicta*.

From a total of 63 samples in 21 locations (see Fig. S1 in the supplemental material), we obtained 100,343 to 114,963 reads and 660 to 1,717 operational taxonomic units (OTUs) from each sample. According to the rarefaction curve (Fig. S2), with increasing sequencing depth, each curve gradually flattened out, indicating that the sequencing amount was reasonable and included most microorganisms in the samples. The number of new OTUs detected by further sequencing was relatively small, and the diversity values among different groups were relatively similar. The samples from Zhanjiang had more OTUs, and the rarefaction curve was monotone increasing, which means that the samples may have contained more microorganisms (Fig. S2).

**FIG 1:**
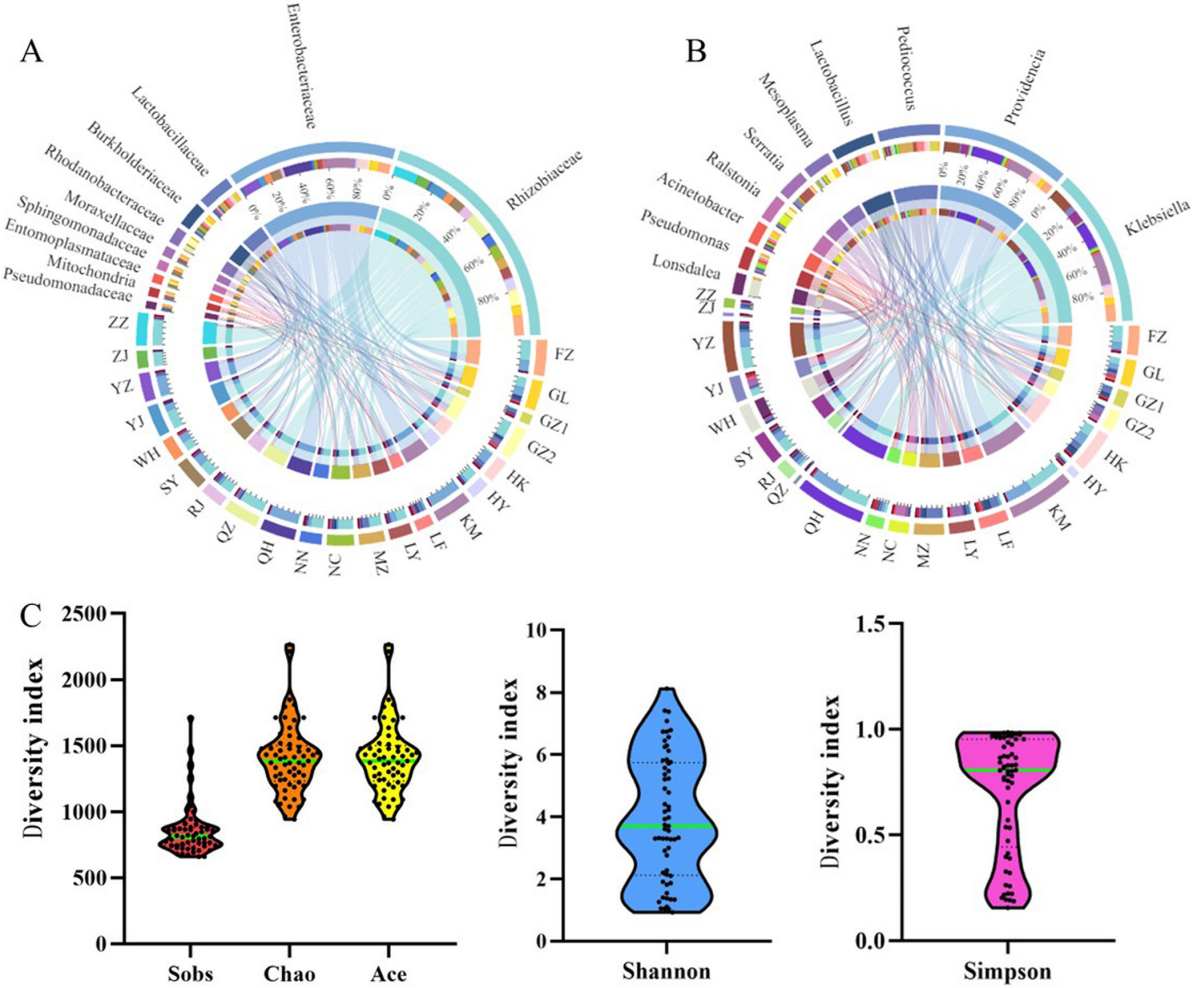
The composition and diversity of gut symbiotic bacteria in *S. invicta.* (A and B) Circos plots showing the distribution of gut symbiotic bacteria at the family level (A) and genus level (B). (C) Sobs, Chao, and ACE indexes of gut symbiotic bacteria in *S. invicta*.

We analyzed the distribution and composition of gut bacteria in all samples according to the family and genus levels (Fig. 1A and B). The Circos plots showed the compositional relationships in the form of sample-gut bacterial connections. The thickness of the corresponding connection reflects the proportion of gut bacteria in the corresponding sample (Fig. 1A and B). Among all OTUs of microorganisms, 32 phyla, 85 classes, 201 orders, 344 families, and 833 genera were found.

The alpha diversity analysis of all populations is outlined in Fig. 1C. Compared to other populations, LY exhibited significantly higher bacterial diversity, reflected in a lower diversity index, such as the Sobs index, Chao index, and ACE index (all *P* values were <0.05) (Fig. S3).

### Population genetic structure and diversity of *S. invicta*.

Overall, 1,260 individuals were successfully genotyped at six microsatellite loci. At the population level, the number of alleles in all populations varied from 1.4 to 6.2, allelic richness ranged from 1.076 to 4.031, and the observed heterozygosity ranged from 0.438 to 1.514. The results indicated that the Yongzhou (YZ) populations exhibited the lowest genetic variation (Table S2). The Bayesian clustering analysis shows that the two genetic clusters showed no relationship with the geographic location of colonies, and individuals from one site were assigned to different genetic clusters (Fig. S5 and S6).

**FIG 2:**
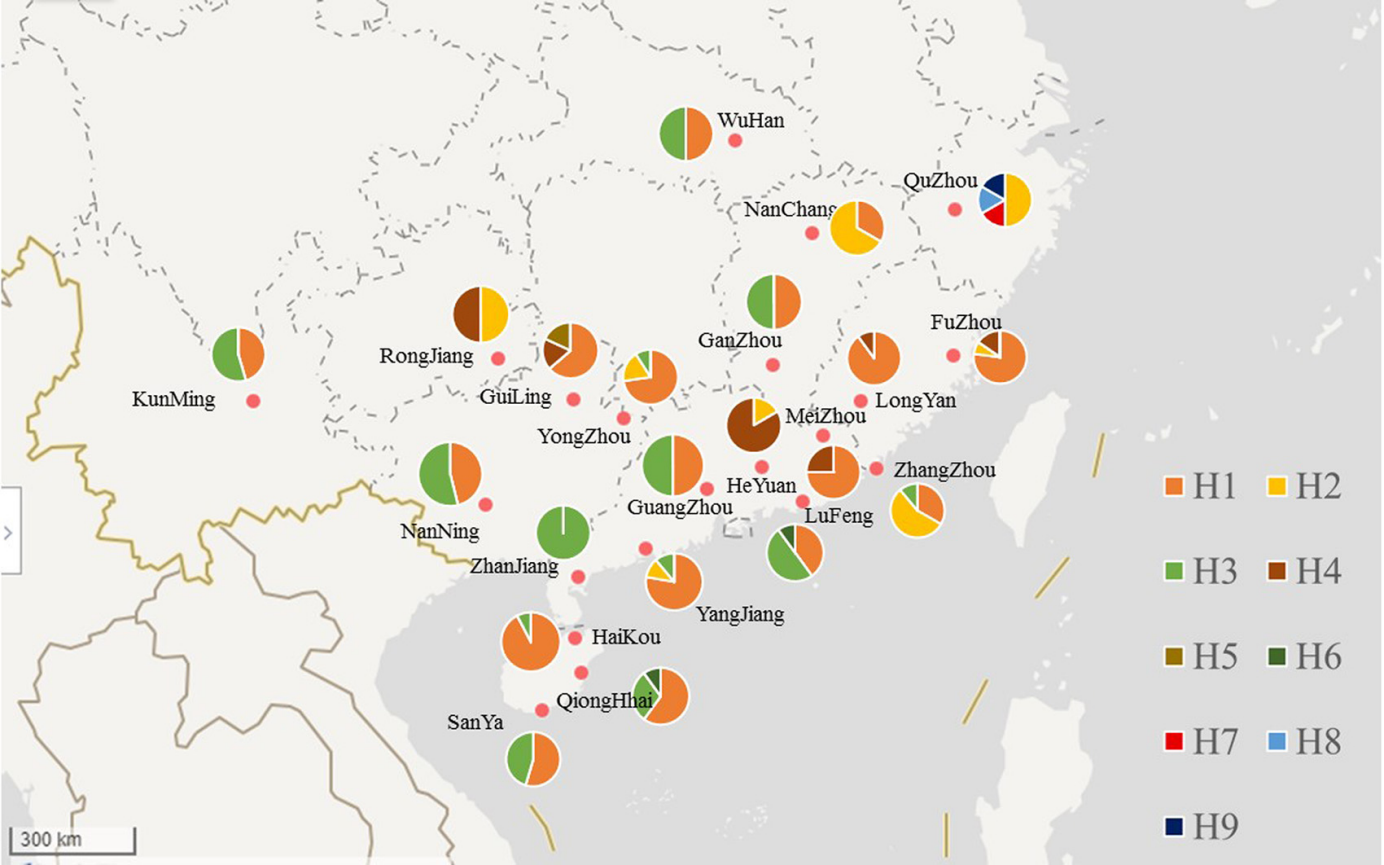
The haplotype distribution pattern of the *S. invicta* populations in 21 regions. The map was generated by https://map.baidu.com/.

A total of 9 COI haplotypes were detected in all the samples: Hap1 to Hap9 (Fig. 2 and Table S1). *S. invicta* haplotype H5 (HM241155.1), haplotype H22 (HM241156.1), haplotype H36 (EU373820.1), haplotype H84 (AY950762.1), and haplotype USA1 (EU373818), which have been published with specific geographic information, were selected from NCBI and used as reference strains. Based on the neighbor-joining (NJ) phylogenetic tree (Fig. S4), Hap1 and Hap4 were identified as *S. invicta* haplotype H22 and haplotype H5, respectively. *S. invicta* haplotype H5 was found in Guangzhou (GZ1), Heyuan (HY), Longyan (LY), Guilin (GL), Rongjiang (RJ), Meizhou (MZ), Nanchang (NC), and Qionghai (QH). *S. invicta* haplotype H22 was found in Yongzhou, Yangjiang (YJ), Sanya (SY), Qionghai, Meizhou, Longyan (LY), Haikou (HK), Guilin (GL), Fuzhou (FZ), etc. (Fig. S4).

We compared the expected heterozygosity (*H_e_*) and observed heterozygosity (*H_o_*) of the *S. invicta* populations in different regions (Table S2) and found that the average *H_o_* was higher than the *H_e_* in all regions, indicating homozygosity loss and a high proportion of heterozygosity. All of the six microsatellite loci exhibited significant deviations from Hardy-Weinberg equilibrium (see Table S2). Therefore, we considered all loci to be independent (37).

The gene flow (*N_m_*) and paired fixed coefficient (*F*_st_) (Table S3) were compared among the different geographic populations of *S. invicta*. The *F*_st_ ranged between 0.037 and 0.616. The lowest *F*_st_ was found between Fuzhou and Meizhou (*F*_st_ = 0.037), and the highest *F_st_* was found between Yongzhou and Zhanjiang (ZJ) or Yangjiang (*F*_st_ = 0.616). The *N_m_* between geographic populations ranged from 0.156 to 6.539, with the maximum between Fuzhou and Meizhou (*N_m_* = 6.539) and the minimum between Yongzhou and Zhanjiang or Yangjiang (*N_m_* = 0.156). The Nei genetic distances of 21 different populations ranged from 0.000 to 2.327 (Table S4), with the maximum between Guilin and Qionghai (Nei’s = 2.327) and the minimum between Yangjiang and Zhanjiang (Nei’s = 0.000).

### Social form of *S. invicta*.

Multiplex PCR and b alleles were used to identify the social form of *S. invicta* from the 21 regions (Table S5). The repetition rate of the results obtained by the two methods reached 96.83%, and only 2 samples were different, indicating that the two identification methods were accurate and that the identification results were reliable. This further indicates these two samples may be polygyny-inducing b′ alleles which need a specific PCR assay which was not performed in our study (38). In general, the ratio of monogyne to polygyne samples for the 63 colony samples in this study was 38:25 (multiplex PCR method) or 40:23 (b allele method), indicating that the monogyne form was dominant in all 21 regions.

### The relationship between host genotype and gut symbiosis.

Genetic differentiation was negatively correlated with geographic distance (*r*^2^ = 0.0667, *P* < 0.001; Fig. S7), while no significant correlation between Nei’s genetic distance and geographic distance was observed (*P* = 0.717; Fig. S8). There was no significant relationship between genetic structure and the diversity of gut bacterial composition of *S. invicta*. Most individuals from the Zhangzhou (ZZ), Lufeng (LF), YZ, GL, Nanning (NN), Kunming (KM), SY, and HK populations were assigned to the same cluster; however, only YZ, GL, NN, KM, SY, and HK populations had the similar pattern of gut bacterial composition. There were significant differences in gut bacterial composition of QH, Wuhan (WH), Quzhou (QZ), NC, Heyuan (HY), YJ, ZJ, and Guangzhou (GZ2) populations, which had similar genetic structures (see Fig. 6 and Fig. S6).

**FIG 3:**
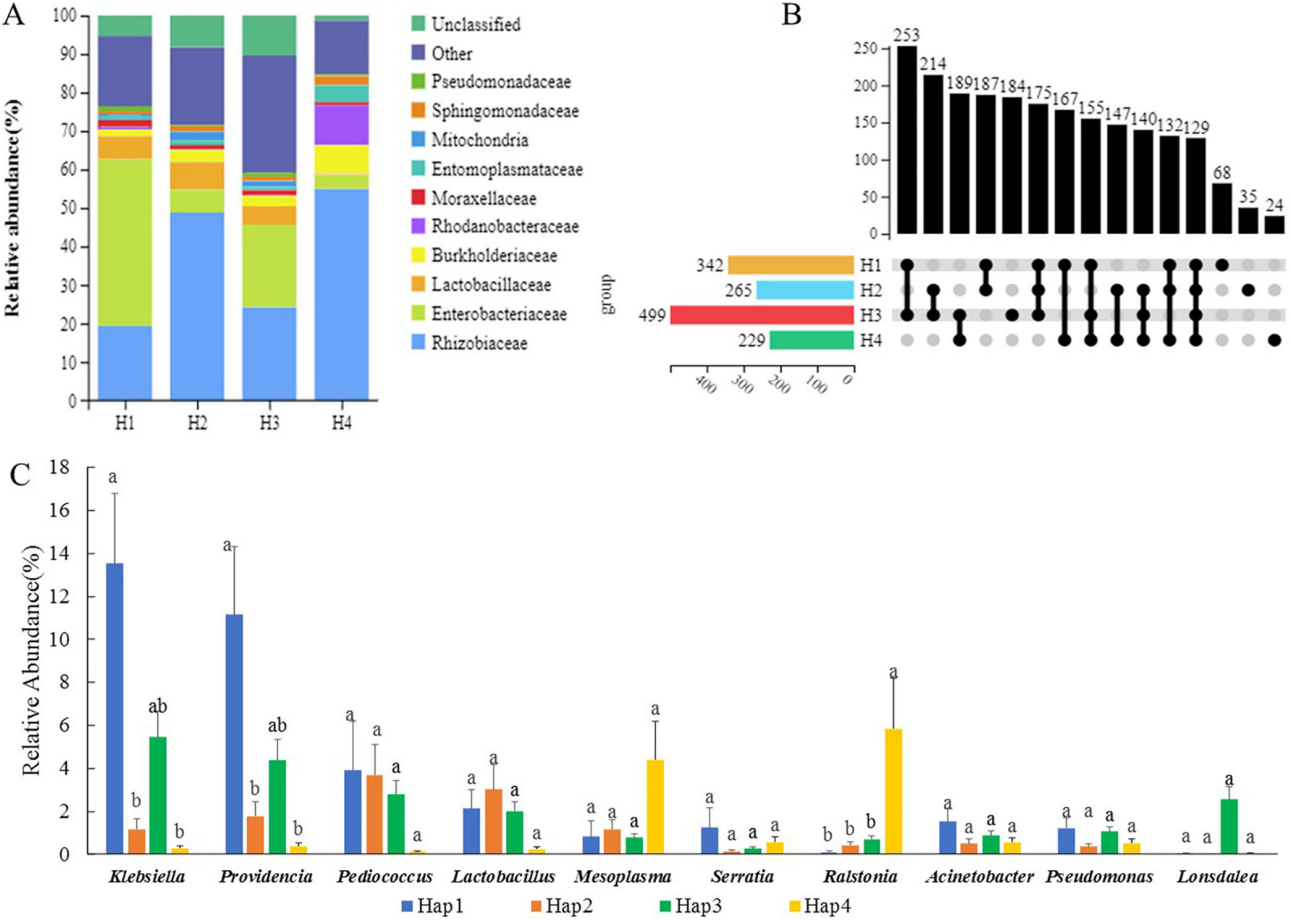
Different symbiotic bacterial communities in the gut of *S. invicta* with different haplotypes. (A) A stack map at the family level for four haplotypes. (B) UpSet of symbiotic bacterial communities in four haplotypes of *S. invicta* (genus level). (C) Comparison of the relative abundances of the main gut symbiotic bacteria in four haplotypes of *S. invicta* at the genus level. Bars with the same letters indicate no significant difference (*P* > 0.05, Mann-Whitney U test).

Several different haplotypes were found in the same nest in some regions. Therefore, we selected the data of samples from the nests in which only a single haplotype (Hap1, Hap2, Hap3, or Hap4) was detected for comparison analysis. There was no significant difference in the Shannon index of gut symbiotic bacterial diversity among the different haplotypes of *S. invicta* (one-way analysis of variance [ANOVA]: *P* = 0.173). The gut symbiotic bacteria of samples from different haplotypes were roughly the same at the family level, but the relative abundance of each bacterial population was different (*P* < 0.05, Fig. 3A). Fifty ant colonies with only a single haplotype (Hap1, Hap2, Hap3, or Hap4) were selected to compare the differences in the number of gut symbiotic bacteria at the genus level by UpSet plot (Fig. 3B). Hap3 had the most genera (499), and Hap4 had the fewest genera (229). The most common genera (253) were distributed between Hap1 and Hap3, with 129 genera found in all four haplotypes (Fig. 3B).

We compared the top 10 most abundant gut symbiotic bacteria (genus level) of these four haplotypes. The proportion of Klebsiella and *Providencia* in Hap1 was significantly higher than that in Hap2 (*P* = 0.014) and Hap4 (*P* = 0.017). The relative abundance of *Ralstonia* in Hap4 was significantly higher than that in the other three haplotypes (*P* < 0.05, Fig. 3C).

The symbiotic bacteria of the four haplotypes consisted of the same genus, but the dominant genus was different. The genera with the highest relative abundance of Hap1 species were Klebsiella (13.545 ± 3.242), *Providencia* (11.148 ± 3.151), and *Pediococcus* (3.907 ± 2.290). The genera with the highest relative abundance of Hap2 species were *Pediococcu*s (3.701 ± 1.399), *Lactobacillus* (3.028 ± 1.144), and *Providencia* (1.785 ± 0.675). The genera with the highest relative abundance of Hap3 species were Klebsiella, *Providencia*, and *Pediococcus.* The genera with the highest relative abundance of Hap4 species were *Ralstonia*, *Mesoplasma*, and *Serratia* (Fig. 3C).

The number of alleles (*N_a_*), effective number of alleles (*N_e_*), Shannon information index (*I*), observed heterozygosity (*H_o_*), expected heterozygosity (*H_e_*), and fixed coefficient *F*_st_ were not correlated with the Shannon index of gut symbiotic bacteria in *S. invicta* (*N_a_*, *r*^2^ = −0.155, *P* = 0.502; *N_e_*, *r*^2^ = −0.192, *P* = 0.404; *I*, *r*^2^ = −0.222, *P* = 0.333; *H_o_*, *r*^2^ = −0.198, *P* = 0.389; *H_e_*, *r*^2^ = −0.199, *P* = 0.388; *F*_st_, *r*^2^ = −0.042, *P* = 0.858).

**FIG 4:**
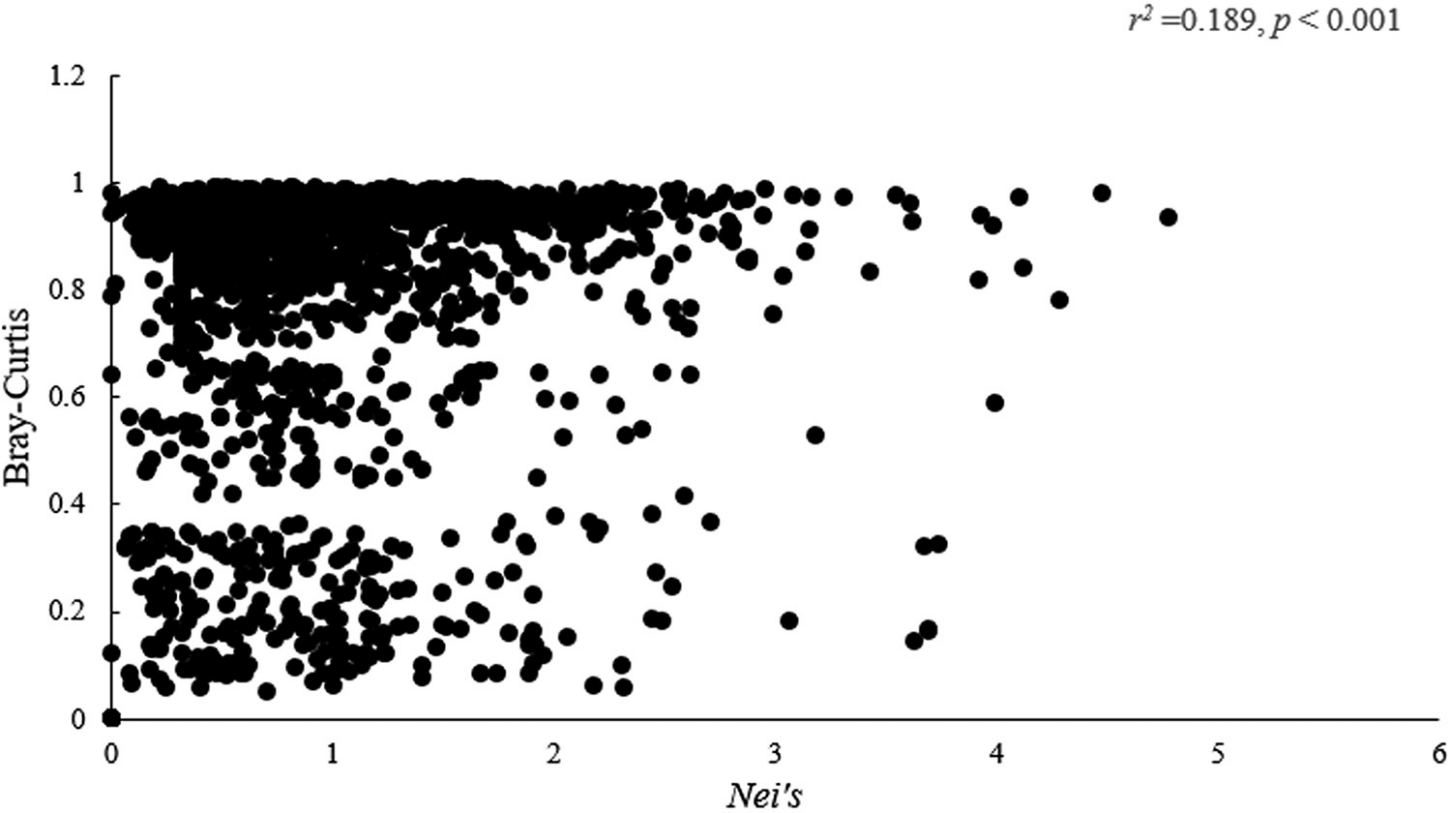
Scatterplot showing correlations between Nei’s genetic distance and the Bray-Curtis dissimilarity matrices of gut symbiotic bacteria *Providencia*, Klebsiella, Acinetobacter, and Pseudomonas in *S. invicta*.

There was a significant positive correlation between Nei’s genetic distance and the Bray-Curtis dissimilarity matrices (*r*^2^ = 0.189, *P* < 0.001) (Fig. 4). There was no correlation between the COI genetic distance and the Bray-Curtis dissimilarity matrices (*r*^2^ = −0.035, *P* = 0.134).

**FIG 5:**
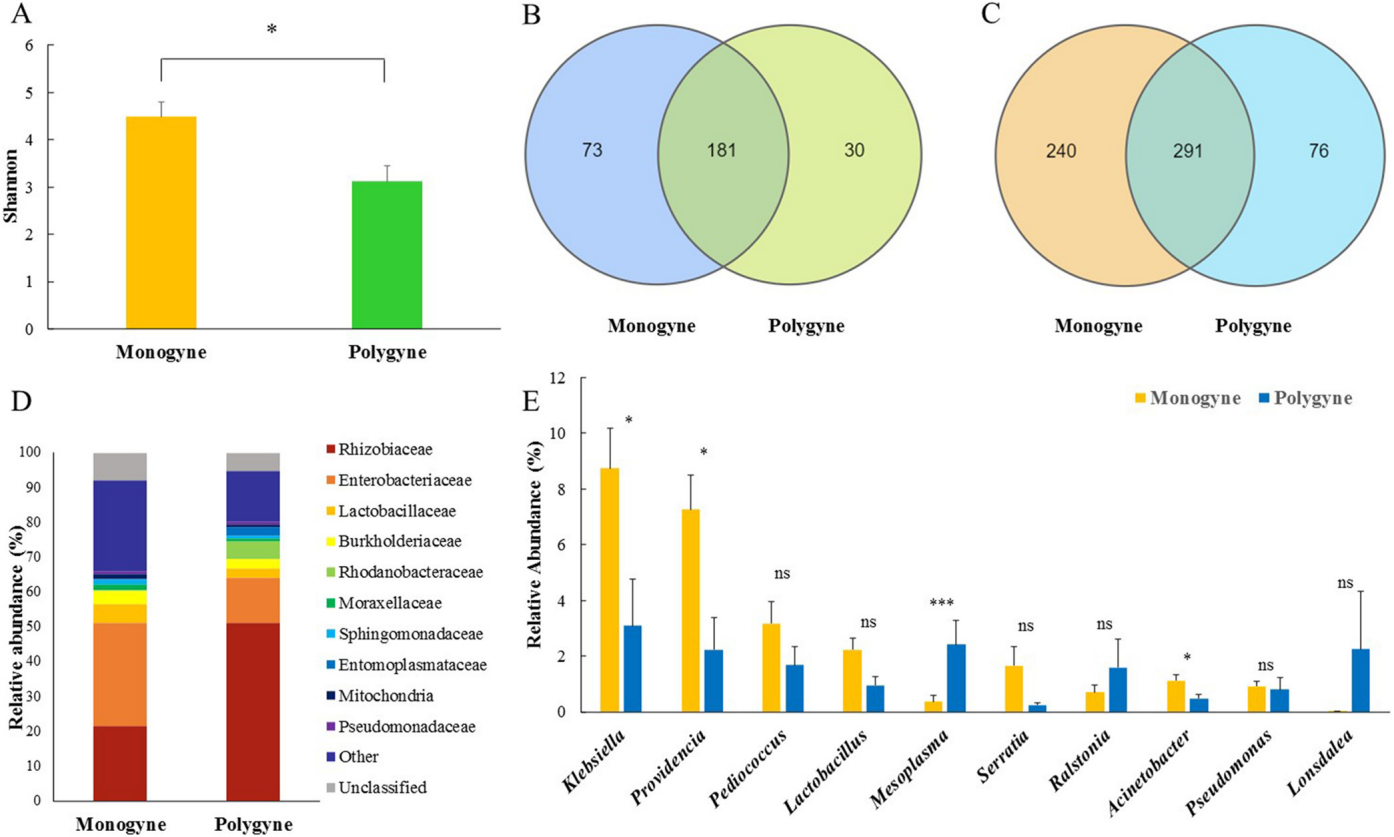
Different symbiotic bacterial communities in the gut of *S. invicta* of different social forms. (A) Shannon index of gut symbiotic bacterial communities of *S. invicta* of different social forms. (B) Species differences between monogyne and polygyne populations in the gut symbiosis of *S. invicta* at the family level. (C) Species differences between monogyne and polygyne populations in the gut symbiosis of *S. invicta* at the genus level. (D) A stack map at the family level for different social forms of *S. invicta*. (E) Comparison of the relative abundances of the main gut symbiotic bacteria in different social forms of *S. invicta* at the genus level. ***, *P* < 0.001; *, *P* < 0.05; ns, no significant difference (*P* > 0.05, Mann-Whitney U test).

The Mann-Whitney U test was performed on the Shannon index data to determine the gut symbiotic bacterial diversity of monogyne and polygyne populations (Fig. 5A), and the results indicated that there were significant differences in the diversity of gut symbiotic bacteria among different social forms (*Z* = 2.556, *P* = 0.011).

There were also differences in the distribution of gut symbiotic species among different social forms of *S. invicta.* At the family level, 254 families were detected in the monogyne group, and 211 families were detected in the polygyne group, of which 181 families were found in both social forms (Fig. 5B). At the genus level, 291 genera were shared by the two social types, 240 genera were unique to the monogyne type, and 76 genera were unique to the polygyne type (Fig. 5C). The Shannon index of gut bacterial diversity was higher in the monogyne population (4.486 ± 0.323) than in the polygyne population (3.121 ± 0.333) (*Z* = −2.556, *P* = 0.011). The main gut symbiotic bacteria corresponding to different social forms were roughly the same at the family level (Fig. 5D), but the relative abundance of each family was different.

We compared the relative abundances of the top 10 populations (genus level) of gut symbiotic bacteria. The Mann-Whitney U test for each population of different social forms revealed significant differences in the relative abundances of Klebsiella (*P* = 0.014, U = 271, *Z* = −2.47), *Providencia* (*P* = 0.005, *P* = 250, *Z* = −2.783), and Acinetobacter (*P* = 0.007, U = 255, *Z* = −2.708) in the two social forms. Moreover, there were extremely significant differences in the relative abundance of *Mesoplasma* between the two social forms (*P* = 0.000). The relative abundance of Klebsiella was significantly higher in the monogyne (8.746 ± 1.422) population than in the polygyne (3.097 ± 1.664) population. The relative abundance of *Providencia* was significantly higher in the monogyne (7.261 ± 1.252) population than in the polygyne (2.236 ± 1.142) population. The relative abundance of Acinetobacter in the monogyne population (1.128 ± 0.195) was higher than that in the polygyne population (0.476 ± 0.135). The relative abundance of *Mesoplasma* was significantly higher in the polygyne population (2.419 ± 0.863) than in the monogyne population (0.366 ± 0.217) (Fig. 5E).

### The relationship between the geographical distribution pattern and gut symbiotic bacteria.

**FIG 6:**
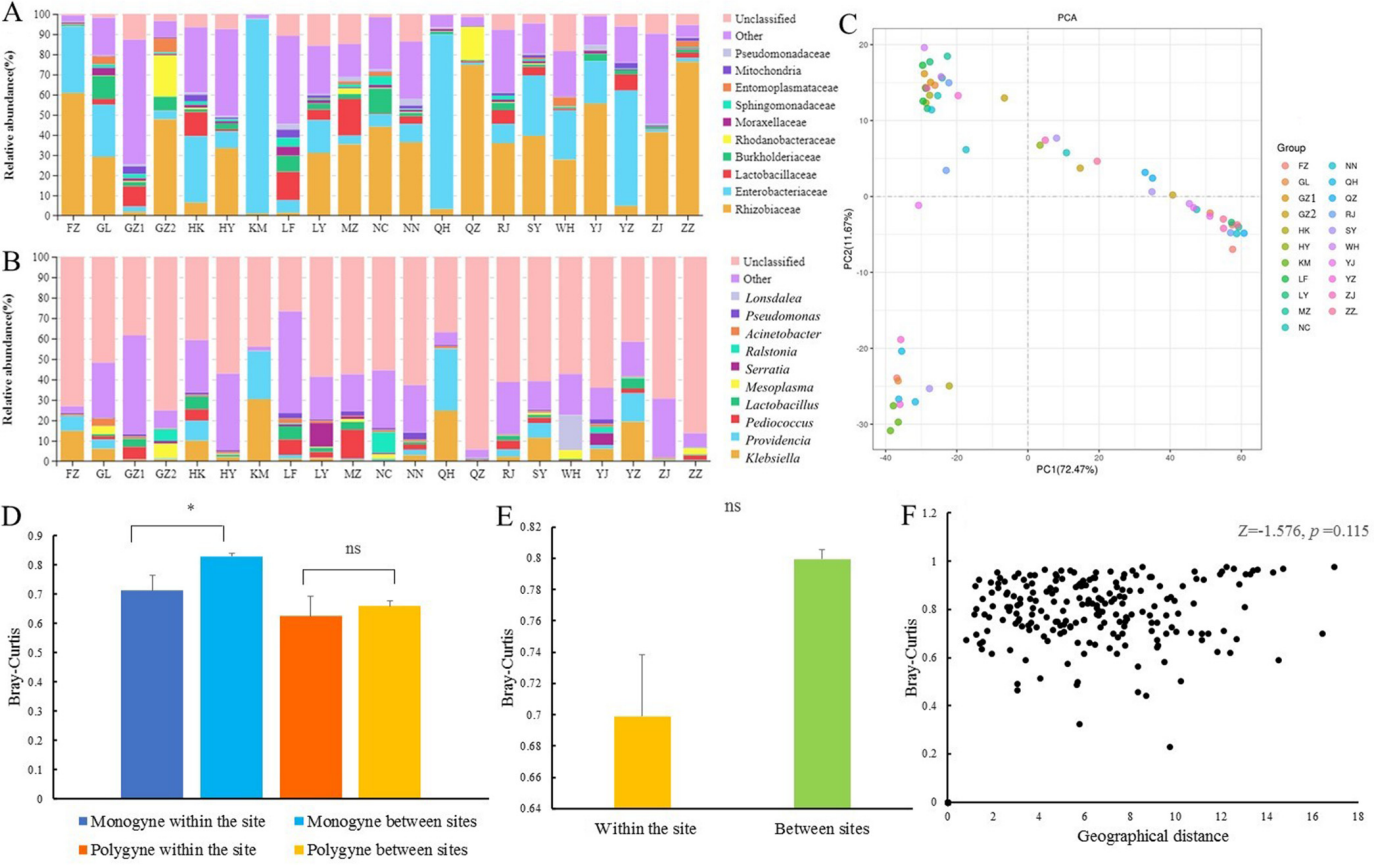
The composition of gut symbiotic bacterial communities of *S. invicta* from different regions and its relationship with geographical distance. (A) A stack map at the family level for different populations of *S. invicta*. (B) A stack map at the genus level for different populations of *S. invicta*. (C) PCA of gut symbiotic bacteria based on OTU abundance information. SY, QH, HK, YJ, ZJ, NN, LF, GZ2, ZZ, HY, MZ, YZ, LY, GL, KM, GZ1, RJ, FZ, NC, QZ, and WH represent Sanya, Qionghai, Haikou, Yangjiang, Zhanjiang, Nanning, Lufeng, Guangzhou, Zhangzhou, Heyuan, Meizhou, Yongzhou, Longyan, Guilin, Kunming, Ganzhou, Rongjiang, Fuzhou, Nanchang, Quzhou, and Wuhan, respectively. (D) Bray-Curtis dissimilarity matrices of gut symbiotic bacteria of *S. invicta* between and within regions with different social forms. (E) Bray-Curtis dissimilarity matrices between and within regions of *S. invicta.* (F) Scatterplots of the geographical distance and Bray-Curtis dissimilarity matrices of gut symbiotic bacteria of *S. invicta.* *, significant difference (*P* < 0.05); ns, no significant difference (*P* > 0.05, Mann-Whitney U test).

The main groups in different regions were similar, but the group distributions were different (Fig. 6A and B and Table S6). According to the diversity analysis of gut symbiotic bacteria from all regions (Fig. S3), the region with the highest Shannon index was Ganzhou (7.08 ± 0.28), whereas Quzhou (1.77 ± 0.6) had the lowest diversity and evenness. According to principal-component analysis (PCA) based on OTU abundance information (Fig. 6C), the first and second principal components accounted for 84.1% of the composition differences among samples from different regions, and the samples were not separated by region.

A Mann-Whitney U test was conducted for the Bray-Curtis dissimilarity matrices of gut symbiotic bacteria between populations from the same region and populations from different areas with different social forms (Fig. 6D). The geographical distance between the sample collection regions for monogyne populations had a significant effect on the Bray-Curtis dissimilarity matrices of gut symbiotic bacteria (*Z* = −3.063, *P* = 0.002). The geographical distance between the sample collection regions for polygyne populations had no significant effect on the Bray-Curtis dissimilarity matrices of gut symbiotic bacteria (*Z* = −0.691, *P* = 0.490).

A Mann-Whitney U test was conducted for the Bray-Curtis dissimilarity matrices of gut symbiotic bacteria between populations from the same region and populations from different areas. The geographical distance between the sample collection regions had no significant effect (*P* = 0.115, U = 61, *Z* = −1.576) on the Bray-Curtis dissimilarity matrices of gut symbiotic bacteria (Fig. 6E).

Spearman correlation analysis was performed on the geographical distances between the sample collection regions and the Bray-Curtis dissimilarity matrices of gut symbiotic bacteria (Fig. 6F). The results indicated that the geographical distance between samples was positively correlated with the Bray-Curtis dissimilarity matrices of gut symbiotic bacteria (*r*^2^ = 0.281, *P* < 0.001).

## DISCUSSION

### Social form affects the distribution and structure of gut microorganisms.

*S. invicta* ants of different social forms significantly differ in their social behaviors and reproductive patterns (39). The average body size of workers from polygyne populations is 16% smaller than that of workers from monogyne populations, and the polygyne workers are less aggressive toward nonnative species (40). This feature makes it easy for monogyne colonies to connect with each other and develop into supercolonies (41).

The Shannon index of gut symbiotic bacterial diversity significantly differed among different social forms, and the monogyne colonies were more diverse. In this study, the abundances of two social forms were not the same (monogyne/polygyne ratio = 3:2), and the samples were randomly collected from different regions; that is, the number of the two social forms collected in the same region was not the same. Therefore, the influence of geographical distribution and other factors could not be completely excluded when comparing different social forms, which may have influenced the analysis results. The gut symbiotic bacterial species of *S. invicta* of different social forms were roughly the same, but the proportions were different. In social insect bees, the structure and abundance of the gut symbiotic bacteria of queens, workers, and drones are different, and workers have richer gut symbiotic bacteria than drones and queens (42). Previous studies also reported caste-dependent diversity of the commensal gut microbial community in ants (43, 44). Gut microorganisms can indirectly affect worker behavior and labor division by regulating vitellogenin and biogenic amines (42, 45). Ants of different social forms differ in their population density, diffusion ability, etc. Polygyne colonies have more workers, a larger colony size, and greater resource consumption and reserves (46), which enable polygyne colonies to obtain more food. Moreover, differences in food acquisition may lead to differences in gut symbiotic bacteria (24, 47). Therefore, it is speculated that social form can affect the distribution and structure of gut microorganisms in *S. invicta* to some extent, but the specific mechanism underlying this effect remains unclear. We hypothesized that there was an interaction mechanism between haplotypes of red imported fire ant and gut symbiotic bacteria, and diet may be the regulatory mechanism between them (24).

### Host genotype and gut microorganisms.

There was no significant difference in the diversity of gut symbiotic bacteria among the different haplotypes of *S. invicta*, but the proportions of dominant bacteria differed. There were no correlations between the Shannon index, Simpson index of gut symbiotic bacteria, and population genetic diversity indexes, including *N_a_*, *N_e_*, *I*, *H_o_*, *H_e_*, and *F*. The Bray-Curtis dissimilarity matrices were positively correlated with Nei’s genetic distance of the host but not with the COI-based genetic distance of the host. STRUCTURE software analysis using microsatellite data suggests that colonies of most of the populations we tested were not uniquely assigned to either of the two genetic clusters. In addition, most workers in our study were heterozygous at microsatellite loci. This may indicate that new queens return to their original nest after mating and that a majority of male reproductive ants come from other nests. These results indicated that the composition of gut microorganisms in *S. invicta* was influenced by the variability of host nuclear genes but not by the mitochondrial genome. This type of association may be achieved by vertical transmission of gut symbiotic bacteria. Insect symbiotic bacteria spread stably through generations of hosts by infecting germ stem cells in larvae and ovaries of female adults, infecting mature oocytes in female adults, and undergoing vertical transmission (48–51). Vertical transmission of insect gut symbiotic bacteria has been found in a variety of insects, such as Pediculus humanus (48). Ants can engage in vertical transmission of gut symbionts, not typically through the germ line but rather through trophallaxis, resulting in ant-ant transmission. Studies on *Cephalotes* ant gut microbiota indicated a high degree of partner fidelity, suggesting the important role of vertical transmission in the evolution and maintenance of the association (19). This method of passing symbiotic bacteria from parent to offspring has many advantages for the host, since important symbionts that are beneficial to the health and physiology of the host are already present in growing larvae (6).

Our analysis showed that the Bray-Curtis dissimilarity matrices of the symbiotic microorganism communities of *S. invicta* in the 21 geographic populations were significantly positively correlated with Nei’s genetic distance of the host. Host genetics have been shown to influence the composition and structure of symbiotic microorganism communities in mammals, insects, plants, and many other organisms (52–54). Martins et al. (55) found a significant correlation between the host (*Solenopsis*) mitochondrial DNA (COI gene) and *Wolbachia* diversity and no significant correlation between the geographic location of the host and the associated *Wolbachia* diversity, strongly suggesting the occurrence of vertical transfer. Su et al. (56) found that the genetic distance of *Wolbachia* was highly significantly correlated with the genetic distance of COI of the host Hylyphantes graminicola, suggesting possible coevolution between *Wolbachia* and the host. These studies suggest that differences in gut microorganisms from different *S. invicta* populations may result from experiencing different environmental conditions, food resources, and biological competitors and predators as well as different genetic backgrounds.

### The geographical distribution pattern influenced the gut symbiotic bacteria.

The Bray-Curtis dissimilarity of gut symbiotic bacteria was significantly positively correlated with the geographical distance of the host *S. invicta*, but the relationship may be affected by the social form. In monogyne colonies, sample geographical distance had a significant effect on the Bray-Curtis dissimilarity matrices of gut symbiotic bacteria, but this effect was not found in polygyne colonies. These results indicated that the geographical distance of the host was closely related to the composition of symbiotic bacteria, and ant colonies with closer geographical distributions had more similar gut symbiotic bacterial compositions. The effect of geographical location on the host gut bacterial community was also verified both in the transplanting bee Apis mellifera *ligustica* and in ants (8, 17, 57). Differences in temperature and food among geographical locations may be factors affecting gut bacteria. There were differences in dominant bacteria, unique bacteria, and microbial gene functions in gut microorganism colonies of the ant Apostichopus japonicus at different growth temperatures (58). After high-temperature treatment, the gut microorganism diversity and composition of the mulberry pyralid Glyphodes pyloalis Walker changed significantly, while the stress resistance of the host increased (58). Jones et al. (59) found that bees exposed to different microenvironments (i.e., inside and outside the hive) and different foods had significantly different gut microbial communities. These results showed an inevitable relationship between the environment of the host and the gut microorganisms.

In conclusion, the Bray-Curtis dissimilarity matrices of gut symbiotic bacteria are significantly positively correlated with the geographical distance of the host ant colony, but this relationship may be affected by the social form of *S. invicta*. The genetic distance of *S. invicta* also influenced the composition of gut symbiotic bacteria. Different haplotypes of red imported fire ants had different gut microorganism compositions, and the diversity indexes were higher in the monogyne colonies than in the polygyne colonies. Both the environment and host genetics influence the composition and abundance of host microbial composition, and these drivers vary spatially.

## MATERIALS AND METHODS

### Sample collection.

All samples of *S. invicta* were collected using an aspirator from May to October 2019 at 21 locations in mainland China. All collection points were separated by at least 100 km. We selected 21 cities in different latitudes south of the Yangtze River as sample collection sites. The sample collection points were in Sanya (SY), Qionghai (QH), Haikou (HK), Yangjiang (YJ), Zhanjiang (ZJ), Nanning (NN), Lufeng (LF), Guangzhou (GZ2), Zhangzhou (ZZ), Heyuan (HY), Meizhou (MZ), Yongzhou (YZ), Longyan (LY), Guilin (GL), Kunming (KM), Ganzhou (GZ1), Rongjiang (RJ), Fuzhou (FZ), Nanchang (NC), Quzhou (QZ), and Wuhan (WH) (see Fig. S1 and Table S7 in the supplemental material). A total of 3 to 5 colonies were collected in each region. All mounds were separated by at least 200 m. After collection, samples were preserved in 100% ethanol and stored at −80°C prior to DNA extraction.

### Gut symbiont DNA extraction and species diversity.

We selected 3 colonies from each region and randomly selected 6 to 20 workers with the same COI haplotype from each colony for gut symbiont DNA extraction. Prior to DNA extraction, we soaked the workers in 75% ethanol for 10 min and washed them with double-distilled water (ddH_2_O) 3 times to remove the bacteria from the body surface. Midguts were picked from the dissected abdomens, and bacterial DNA was extracted with a DP302 bacterial genome DNA extraction kit (TianGen Biology Co., Ltd., Beijing, China). Each sample contained the pooled guts of 10 workers from the same colony. To prevent contamination from the DNA extraction kit and laboratory reagents (60), we included 3 negative controls with sterile distilled water originating from the DNA extraction step.

The V3+V4 parts of 16S rRNA genes were amplified by primers 341F and 806R (341F, CCTACGGGNGGCWGCAG; 806R, GGACTACHVGGGTATCTAAT). The first PCR was performed in a 50-μL volume containing 10 μL 5× Q5@ reaction buffer, 10 μL 5× Q5@ high-GC enhancer, 1.5 μL 2.5 mM deoxynucleoside triphosphates (dNTPs), 1.5 μL primer F (10 μΜ), 1.5 μL primer R (10 μM), 0.2 μL Q5@ high-fidelity DNA polymerase, the template 2 μL (50 ng), and H_2_O up to 50 μL. The cycling conditions for amplification were as follows: 95°C at 5 min followed by 30 cycles of 95°C for 1 min, 60°C for 1 min, and 72°C for 1 min, with a final extension at 72°C for 7 min.

Amplicons were extracted from 2% agarose gels and purified using the AxyPrep DNA gel extraction kit (Axygen Biosciences, Union City, CA, USA) according to the manufacturer’s instructions and quantified using a QuantiFluor-ST (Promega, USA). The purified amplicons were then pooled in an equimolar fashion and subjected to paired-end sequencing (2 × 250) on an Illumina HiSeq 2500 platform according to standard protocols. For each sample, more than 50,000 reads were obtained. After sequencing, the data were filtered using FASTP (https://github.com/OpenGene/fastp) to remove reads containing more than 10% unresolved nucleotides (N) and reads containing less than 80% bases with a Q value of >20. Paired-end clean reads were merged as raw tags using FLSAH (v 1.2.11) with a minimum overlap of 10 bp and mismatch error rates of 2%. Noisy sequences of raw tags were filtered by the QIIME (V1.9.1) pipeline under specific filtering conditions (61) to obtain high-quality clean tags. Clean tags were searched against the reference database (http://drive5.com/uchime/uchime_download.html) to perform reference-based chimera checking using the UCHIME algorithm (http://www.drive5.com/usearch/manual/uchime_algo.html). All chimeric tags were removed, and effective tags were finally obtained for further analysis. To obtain unique tags and to determine the number of tags in the data set, the data set was subjected to redundancy treatment using Mothur software (v. 1.27.0) (62). Moreover, rarefaction curves were calculated by Mothur for all samples to evaluate the sequencing saturation. The representative sequences were classified into organisms by a naive Bayesian model using the RDP classifier (63) (version 2.2) based on the SILVA (64) database (https://www.arb-silva.de/), with the confidence threshold values ranging from 0.8 to 1. The tags were clustered into operational taxonomic units (OTUs) at ≥97% similarity using the UPARSE (65) pipeline. The tag sequence with the highest abundance was selected as a representative sequence within each cluster. The species annotations and abundance information of the OTUs were used to generate OTU abundance profiles for all samples. We also identified and removed OTUs that were more abundant in negative controls than real samples by using the decontam package in R (v3.4.3) (66). Principal-component analysis (PCA) was performed based on the OTU abundance information of samples to explore the compositional distance relationship between samples from different regions. Based on the OTU abundance of samples, alpha diversity indexes, including the Chao1, ACE, Shannon, Sobs, and Simpson indexes, were calculated with QIIME’s 1.9.1 alpha_diversity script using the default settings (67). Based on the OTU abundance and species classification, R: vegan was used to calculate the Bray-Curtis dissimilarity matrix index. Bioinformatic analysis was performed using Omicsmart, a dynamic real-time interactive online platform for data analysis (http://www.omicsmart.com).

### Genotype of fire ant determined by microsatellite genotyping.

We selected 3 colonies from each region and randomly selected 20 workers from each colony for DNA extraction and PCR amplification. We amplified six microsatellite loci (Table S8) developed by Krieger and Keller (68). Primers were synthesized by Sangon Biotech Co., Ltd. (Shanghai, China). The DNA of each single whole red imported fire ant was amplified by PerfectStart *Taq* DNA polymerase (TransGen Biotech, China). Single-primer PCR or multiplex PCR was performed according to the allele fragment size.

Sol-20 single-primer PCR was performed in a 20-μL volume containing 3.5 μL of DNA, 0.5 μL of each primer (10 mmol/L), 2.5 μL of *Taq* buffer, 2 μL of dNTPs, 0.5 μL of polymerase, and 10.5 μL of ddH_2_O. The cycling conditions for amplification were as follows: 3 min at 94°C followed by 33 cycles of 30 s at 94°C, 30 s at 50°C, and 1 min at 72°C, and a final extension of 5 min at 72°C. Sol-6 and Sol-55 multiplex PCRs were performed in a 20-μL volume containing 3.5 μL of DNA, 0.5 μL of each primer (10 mmol/L), 2.5 μL of *Taq* buffer, 2 μL of dNTPs, 0.5 μL of polymerase, and 9.5 μL of ddH_2_O. The cycling conditions for amplification were as follows: 3 min at 94°C followed by 33 cycles of 30 s at 94°C, 30 s at 53°C, and 1 min at 72°C, with a final extension of 5 min at 72°C. Sol-11, Sol-42, and Sol-49 multiplex PCRs were performed in a 20-μL volume containing 3.5 μL of DNA, 0.5 μL of each primer (10 mmol/L), 2.5 μL of *Taq* buffer, 2 μL of dNTPs, 0.5 μL of polymerase, and 8.5 μL of ddH_2_O. The cycling conditions for amplification were as follows: 3 min at 94°C followed by 33 cycles of 30 s at 94°C, 30 s at 60°C, and 1 min at 72°C, and an extension of 5 min at 72°C.

The PCR-amplified products were confirmed by agarose gel electrophoresis in a 2% agarose gel run at 120 V for 20 min and then sent to Sangon Biotech Co., Ltd. (Shanghai, China), for short tandem repeat (STR) sequencing. Population genetic parameters, including the observed number of alleles (*N_a_*), the effective number of alleles (*N_e_*), Shannon’s information index (*I*), observed heterozygosity (*H_o_*), expected heterozygosity (*H_e_*), the percentage of polymorphic loci (*P*), Nei’s genetic diversity (Nei’s), the inbreeding coefficient in the population (*F*_is_), and the differentiation coefficient among populations (*F*_st_), were then analyzed using GenAlex 6.5.

The Hardy-Weinberg equilibrium for each locus and linkage disequilibrium (LD) for each pair of loci were tested by GENEPOP version 4.5.1 (69). To determine the genetic relationships among populations, each colony was considered a subpopulation in each geographic site and was subjected to an independent simulation run by STRUCTURE software based on the admixture model and an allele frequency-correlated model, with different genotypes of individuals allocated to different genetic clusters (70). Here, we ran all individuals genotyped at the population level, taking the first individual genotyped per colony. We performed 20 times for each value of *K*. Each run included a 1 × 10^5^ burn-in period followed by 1 × 10^5^ iterations of the Markov chain Monte Carlo (MCMC) method. The most likely number of genetic clusters was evaluated using the ΔK method (71) implemented in Structure Harvester v.0.6.8 (72).

The correlation coefficients between *F*_st_/(1 − *F*_st_) or Nei’s genetic distance and the natural log of the geographic distance (kilometers) were run using GENEPOP version 4.5.1 with Mantel tests (69).

### COI gene amplification.

Another 20 workers from each of the 3 colonies from each region were randomly selected for DNA extraction (Table S7). Total DNA from each worker (the whole body) was extracted using the TIANamp genomic DNA kit DP304 (TianGen Biology Co., Ltd., Beijing, China).

We selected 6 workers randomly for PCR amplification of their DNA. A 950-bp fragment of the COI gene was amplified using primers (C1-J-2195, 5′-TTGATTTTTTGGTCATCCAGAAGT-3′; DDS-COII-4, 5′-TAAGATGGTTAATGAAGAGTAG-3′) synthesized by Sangon Biotech Co., Ltd. (Shanghai, China), and PerfectStart *Taq* DNA polymerase (TransGen Biotech, China). PCR was performed in a 20-μL volume containing 3.5 μL of DNA, 0.5 μL of each primer (10 mmol/L), 2.5 μL of *Taq* buffer, 2 μL of dNTPs, 0.5 μL of polymerase, and 10.5 μL of ddH_2_O. The cycling conditions for amplification were as follows: 3 min at 94°C followed by 35 cycles of 30 s at 94°C, 30 s at 55°C, and 1 min at 72°C, with a final extension of 5 min at 72°C. The PCR-amplified products were confirmed by agarose gel electrophoresis in a 1% agarose gel run at 110 V for 20 min and then sent to Sangon Biotech Co., Ltd. (Shanghai, China), for sequencing.

We aligned all sequences using MEGA 6.0 software. COI genetic distance was calculated by MEGA 6.0. A population phylogenetic tree was reconstructed using the neighbor-joining method in MEGA 6.0 based on genetic distances. Haplotype diversity (*H_d_*), the average number of differences (*K*), and nucleotide diversity (π) were calculated for each geographic population using DnaSP 5.0. Spearman correlation analysis was performed on the correlation of COI genetic distance, Nei’s genetic distance, and the Bray-Curtis dissimilarity matrices using SPSS v22.0.

### Detection of social form on the basis of Gp-9. (i) Multiplex PCR.

We selected 3 colonies from each region and randomly selected 10 workers from each colony for DNA extraction and PCR amplification (Table S7). Referring to the method of Valles and Porter (73), we selected 4 primers (Table S9) synthesized by Sangon Biotech Co., Ltd. (Shanghai, China). PerfectStart *Taq* DNA polymerase (TransGen Biotech, China) was used for multiplex PCR amplification. The PCR mixtures each contained 2 μL of DNA, 0.5 μL of each primer, 2.5 μL of *Taq* buffer, 0.5 μL of polymerase, 2 μL of dNTPs, and 16 μL of H_2_O. The cycling conditions for amplification were as follows: 3 min at 94°C followed by 33 cycles of 30 s at 94°C, 30 s at 53°C, and 1 min at 72°C, and then a final extension of 5 min at 72°C. The PCR-amplified products were separated by agarose gel electrophoresis in a 2% agarose gel run at 120 V for 30 min. The social form of a sample was identified based on the number of agarose gel bands. Two single bands were observed in the polygyne samples, and one band was observed in the monogyne samples.

### (ii) *Gp-9^b^* allele PCR.

We selected 3 colonies from each region and randomly selected 10 workers from each colony for DNA extraction and PCR amplification (Table S7). Referring to the method of Mescher et al. (38), we used primers Gp9 b.for (5′-TCGCCGATTCTAACAAAGCT-3′) and Gp9 b.prime.for (5′-TCGCCGATTCTAACGAAGCT-3′) synthesized by Sangon Biotech Co., Ltd. (Shanghai, China). TianGen 2× *Taq* PCR mix (TianGen Biotech [Beijing] Co., Ltd.) was used for PCR amplification, and the reaction mixtures contained 3 μL of DNA, 1 μL of each primer, 12.5 μL of mix, and 7.5 μL of H_2_O. The cycling conditions for amplification were as follows: 2 min at 94°C followed by 35 cycles of 15 s at 94°C, 15 s at 51°C, and 15 s at 68°C, with a final extension of 5 min at 65°C. The PCR-amplified products were separated by agarose gel electrophoresis in a 2% agarose gel run at 120 V for 30 min. The social form of each sample was identified based on the number of agarose gel bands. A single band was observed in the polygyne samples, and no band was observed in the monogyne samples.

### Statistical analyses.

The normality of the data was assessed using the Shapiro-Wilk test in SPSS 22.0. The Kruskal-Wallis one-way ANOVA or Mann-Whitney U test was performed to compare the differences in the diversity index of gut symbiotic bacteria of different populations, haplotypes, and social forms and the Bray-Curtis dissimilarity matrices of gut symbiotic bacteria of *S. invicta* between different social forms and regions of *S. invicta.*

### Data availability.

Data archives for sequences of COI (accession numbers ON130758 to ON130851) and 16S rRNA (accession number PRJNA816559) have been deposited in NCBI GenBank.
