# Supplementary material for: Gut Microbiome Composition of the Fire Ant Solenopsis invicta: an Integrated Analysis of Host Genotype and Geographical Distribution
Source: Microbiol Spectr. 2023 Jan 5;11(1):e03585-22. doi: 10.1128/spectrum.03585-22 (PMC9927370; doi:10.1128/spectrum.03585-22)
Supplement: Supplemental file 1 — Fig. S1 to S8 and Tables S1 to S9. Download spectrum.03585-22-s0001.pdf, PDF file, 0.7 MB [file spectrum.03585-22-s0001.pdf]

Gut microbiome composition of the fire ant *Solenopsis invicta*: an integrated analysis of host genotype and geographical distribution

Qian Xiao[1#], Lei Wang[1#], Siqi Chen[1], Chunyan Zheng[1], Yongyue Lu[1], Yijuan Xu[1*]

1 Red Imported Fire Ant Research Center, South China Agricultural University, Guangzhou 510642 China

*Correspondence: Yijuan Xu, Red Imported Fire Ant Research Center, South China Agricultural University, Guangzhou 510642 China. Email: xuyijuan@yahoo.com

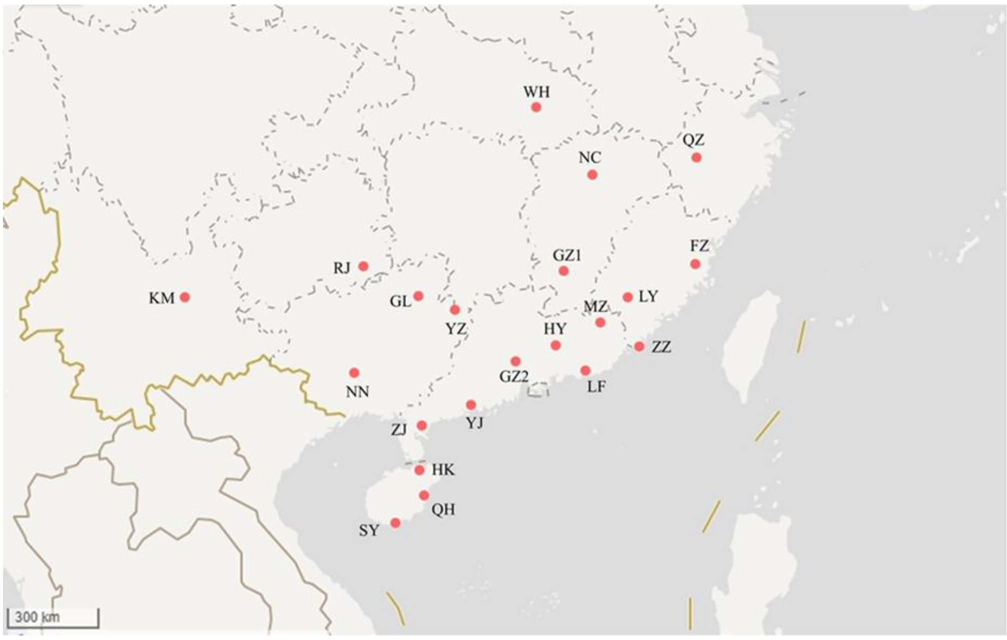

Figure S1 Location of the 21 populations of *S. invicta* examined in this study. The map was generated by https://map.baidu.com/.

SY、QH、HK、YJ、ZJ、NN、LF、GZ2、ZZ、HY、MZ、YZ、LY、GL、KM、GZ1、RJ、FZ、NC、QZ、WH represents Sanya, Qionghai, Haikou, Yangjiang, Zhanjiang, Nanning, Lufeng, Guangzhou, Zhangzhou, Heyuan, Meizhou, Yongzhou, Longyan, Guilin, Kunming, Ganzhou, Rongjiang, Fuzhou, Nanchang, Quzhou, and Wuhan respectively.

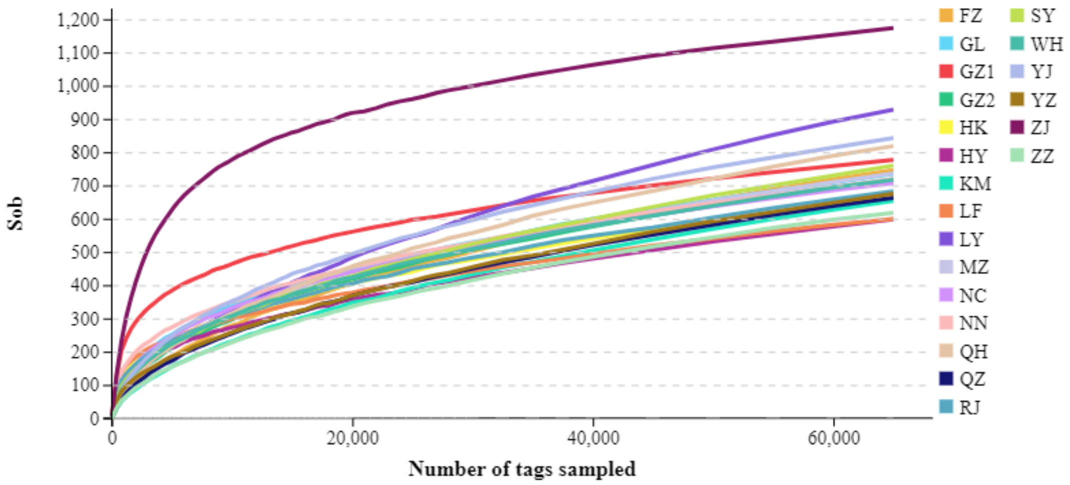

Figure S2 The dilution curve

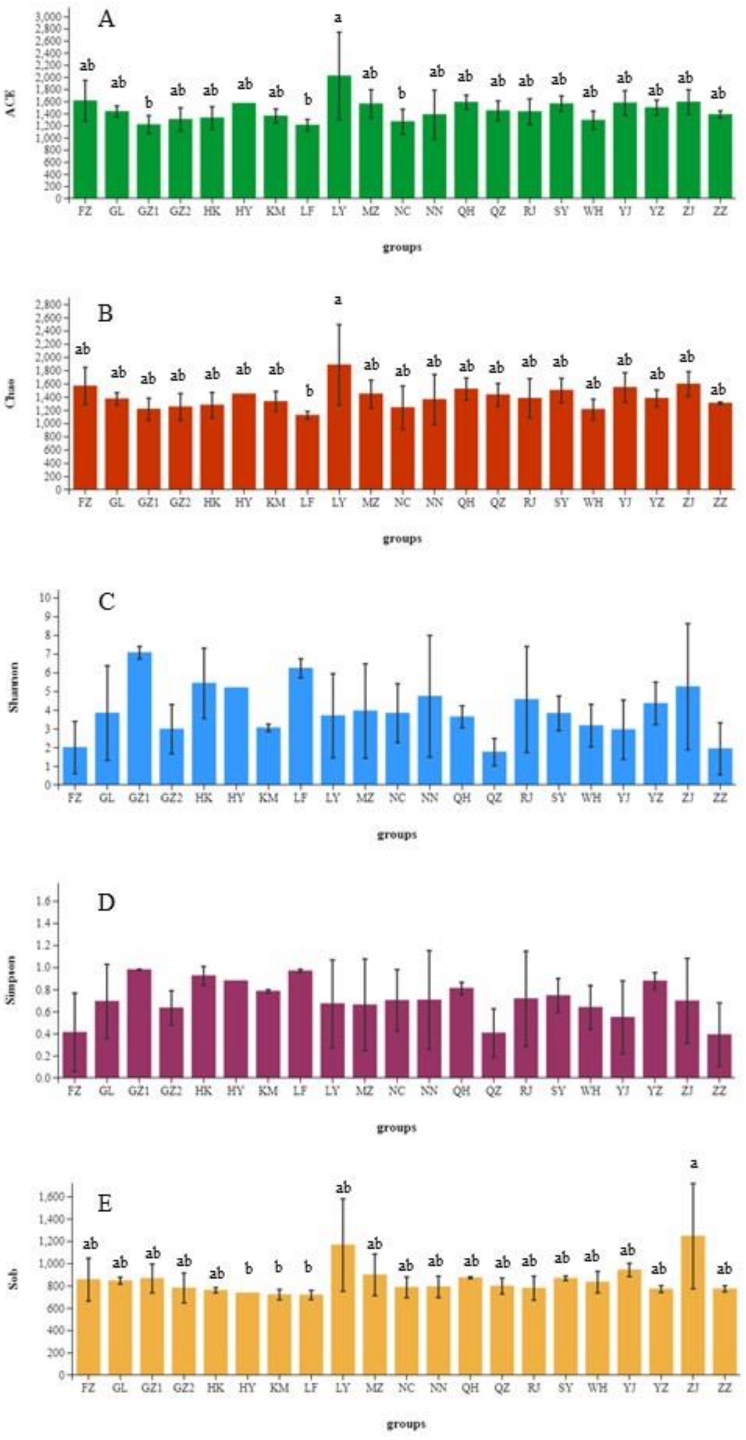

Figure S3 Ace (A), Chao (B), Shannon (C), Simpson (D) and Sobs (E) indexes of gut symbiotic bacteria in *S. invicta* from different population. Bars (mean ± SE) with the same letter indicate no significant difference (Mann Whitney, P>0.05).

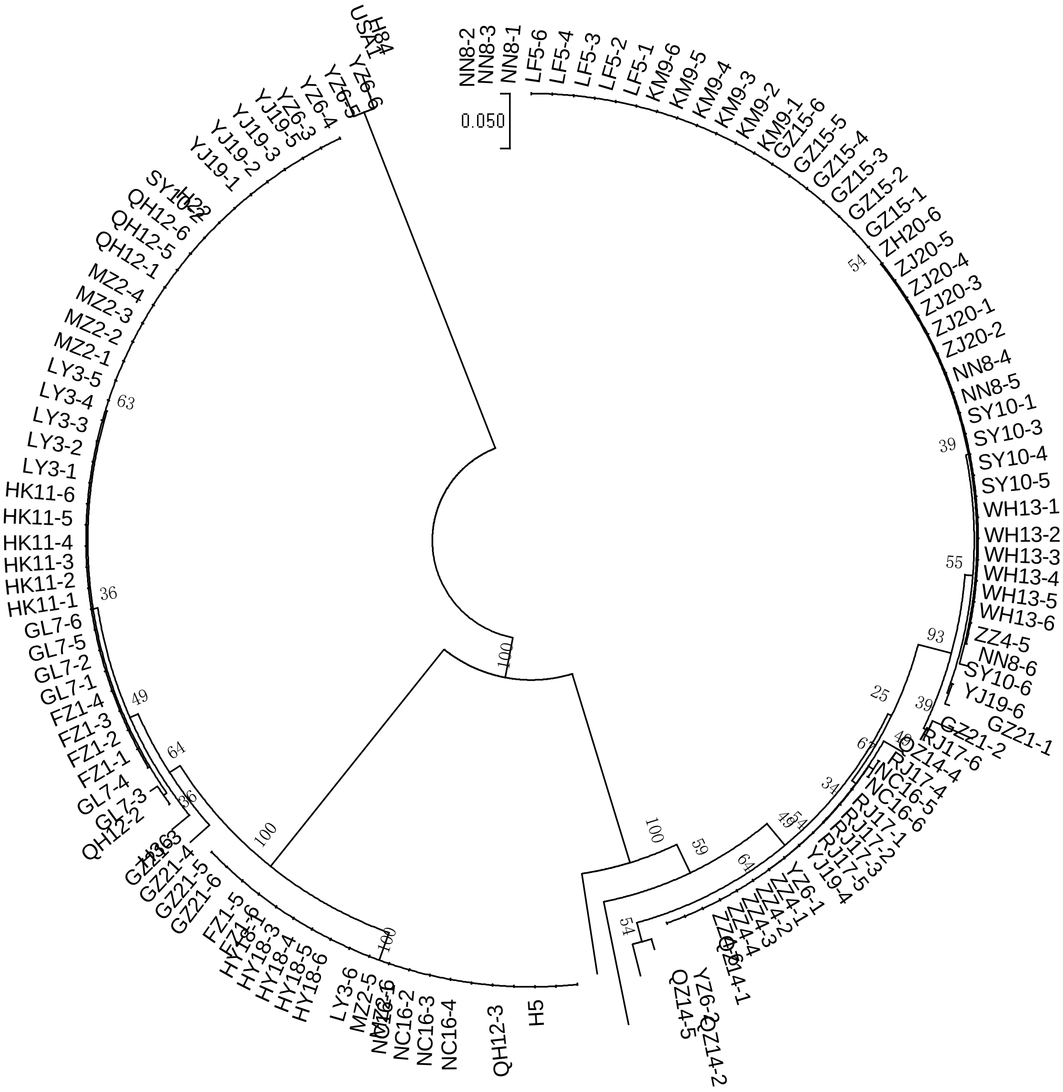

Figure S4 Phylogenetic tree of NJ method based on *CO I* sequence

Table S1 Distribution of haplotypes of red imported fire ants in different geographic populations

Colonies	The sequence number	Haplotypes (H)	Haplotypes diversity (Hd±SD)	Average number of nucleotide differences (K)	Nucleotide diversity(π)
Fuzhou	13	H1(10)，H2(1)，H4(2)	0.52564±0.153	7.92308	0.16170
Meizhou	12	H1(9)，H4(3)	0.409±0.133	1.22727	0.02505
Longyan	10	H1(9)，H4(1)	0.200±0.154	0.600	0.01224
Zhangzhou	9	H1(3)，H2(5)，H3(1)	0.63889±0.091	13.333	0.27211
Lufeng	10	H1(4)，H3(5)，H6(1)	0.644±0.101	13.95556	0.28481
Yongzhou	11	H1(8)，H2(2)，H3(1)	0.47273±0.175	11.30909	0.23080
Guilin	11	H1(7)，H4(2)，H5(2)	0.58182±0.154	1.16364	0.02375
Nanning	13	H1(6)，H3(7)	0.53846±0.078	13.46154	0.27473
Kunming	11	H1(5)，H3(6)	0.54545±0.072	13.63636	0.27829
Sanya	11	H1(6)，H3(5)	0.54545±0.072	13.63636	0.27829
Haikou	13	H1(12)，H3(1)	0.16667±0.134	4.16667	0.08503
Qionghai	13	H1(9)，H4(3)，H6(1)	0.50000±0.143	5.15385	0.10518
Wuhan	12	H1(6)，H3(6)	0.54545±0.128	13.63636	0.46648
Quzhou	6	H2(3), H7(1), H8(1), H9(1)	0.80000±0.172	13.13333	0.26803
Ganzhou	14	H1(7)，H3(7)	0.53846±0.076	13.46154	0.27473
Nanchang	6	H2(2)，H4(4)	0.53333±0.096	13.33333	0.27211
Rongjiang	12	H4(6)，H2(6)	0.54545±0.104	13.63636	0.27829
Heyuan	6	H2(1)，H4(5)	0.33333±0.122	8.33333	0.17007
Yangjiang	9	H1(7)，H2(1)，H3(1)	0.41667±0.159	10.00000	0.20408
Zhanjiang	6	H3(6)	0.00000	0.00000	0.00000
Guangzhou	6	H3(2)，H4(4)	0.53333±0.122	13.86667	0.49169
Total	211	H1(108)、H2(15)、H3(48)、H4(24)、H5(2)、H6(2)、H7(1)、H8(1)、H9(1)	0.663±0.025	12.147	0.24790

Table S2 Genetic diversity of 21 geographic populations of red imported fire ants at five microsatellite loci

Colonies	*Na*	*Ne*	*I*	*Ho*	*He*	*uHe*	*F*	*P*	*PHWE*
Sanya	4.200	2.917	0.981	0.600	0.455	0.464	-0.339	60.00%	All p <0.05
Qionghai	2.600	1.865	0.706	0.600	0.391	0.397	-0.584	60.00%	All p <0.05
Haikou	3.400	2.037	0.819	0.600	0.420	0.425	-0.435	60.00%	All p <0.05
Yangjiang	2.600	1.534	0.624	0.600	0.362	0.369	-0.671	60.00%	All p <0.05
Zhanjiang	2.600	1.534	0.624	0.600	0.362	0.369	-0.671	60.00%	All p <0.05
Nanning	4.200	2.282	0.888	0.600	0.427	0.434	-0.428	60.00%	All p <0.05 expect for locus Sol-42
Lufeng	2.200	1.539	0.617	0.600	0.361	0.366	-0.681	60.00%	All p <0.05 expect for locus Sol-55
Guangzhou	4.800	2.918	1.024	0.600	0.462	0.474	-0.313	60.00%	All p <0.05 expect for locus Sol-42
Zhangzhou	6.200	4.031	1.514	1.000	0.735	0.748	-0.373	100.00%	All p <0.05 expect for locus Sol-55, 42
Heyuan	3.600	2.202	0.856	0.600	0.432	0.438	-0.395	60.00%	All p <0.05
Meizhou	6.200	3.424	1.368	1.000	0.700	0.714	-0.439	100.00%	All p <0.05 expect for locus Sol-11
Yongzhou	1.400	1.076	0.438	0.400	0.251	0.255	-0.594	40.00%	All p <0.05 expect for locus Sol-55
Longyan	4.000	2.560	0.977	0.800	0.533	0.638	-0.539	80.00%	All p <0.05 expect for locus Sol-6
Guilin	4.200	2.862	1.070	0.800	0.554	0.562	-0.461	80.00%	All p <0.05
Kunming	5.000	3.409	1.342	1.000	0.701	0.718	-0.431	100.00%	All p <0.05 expect for locus Sol-49
Ganzhou	2.400	1.731	0.692	0.600	0.389	0.399	-0.550	60.00%	All p <0.05
Rongjiang	3.000	2.078	0.811	0.600	0.419	0.431	-0.443	60.00%	All p <0.05 expect for locus Sol-20, 42, 49
Fuzhou	5.600	3.353	1.346	1.000	0.698	0.713	-0.436	100.00%	All p <0.05 expect for locus Sol-55
Nanchang	3.000	2.009	0.790	0.600	0.420	0.425	-0.429	60.00%	All p <0.05
Quzhou	2.800	1.828	0.736	0.600	0.401	0.408	-0.502	60.00%	All p <0.05
Wuhan	3.400	2.074	0.829	0.600	0.421	0.431	-0.433	60.00%	All p <0.05
Average	3.686	2.346	0.907	0.686	0.471	0.485	-0.475	68.57%	

Na: the number of alleles; *Ne*: the effective number of alleles; *Ho*: the observed heterozygosity; *He*: the expected heterozygosity; *I*: Shannon-Weiner index; *F*: Fixation Index; *P*: Percentage of Polymorphic Loci. PHWE: shows the *p* value for the test of Hardy-Weinberg equilibrium.

Table S3 *Nm*(lower triangle) and *Fst*(upper triangle) distance between 21 geographic populations of red imported fire ants

Colonies	Fuzhou	Meizhou	Longyan	Zhangzhou	Lufeng	Yongzhou	Guilin	Nanning	Kunming	Sanya	Haikou	Qionghai	Wuhan	Quzhou	Ganzhou	Nanchang	Rongjiang	Heyuan	Yangjiang	Zhanjiang	Guangzhou
Fuzhou		0.037	0.185	0.068	0.332	0.441	0.193	0.310	0.090	0.299	0.317	0.280	0.274	0.272	0.313	0.285	0.281	0.299	0.296	0.296	0.273
Meizhou	0.009		0.176	0.061	0.325	0.443	0.206	0.301	0.081	0.290	0.308	0.262	0.264	0.269	0.312	0.279	0.268	0.293	0.285	0.285	0.268
Longyan	0.038	0.036		0.204	0.431	0.524	0.334	0.406	0.216	0.397	0.403	0.373	0.360	0.360	0.374	0.371	0.368	0.375	0.401	0.401	0.363
Zhangzhou	0.016	0.014	0.041		0.265	0.377	0.165	0.257	0.054	0.270	0.270	0.310	0.295	0.301	0.333	0.298	0.306	0.306	0.303	0.303	0.295
Lufeng	0.055	0.055	0.061	0.049		0.561	0.419	0.413	0.309	0.433	0.432	0.481	0.473	0.486	0.524	0.472	0.490	0.497	0.471	0.471	0.475
Yongzhou	0.062	0.062	0.062	0.059	0.062		0.478	0.553	0.413	0.547	0.559	0.603	0.584	0.595	0.595	0.582	0.590	0.588	0.616	0.616	0.576
Guilin	0.039	0.041	0.056	0.034	0.061	0.062		0.400	0.179	0.391	0.401	0.446	0.432	0.441	0.445	0.427	0.427	0.431	0.442	0.442	0.418
Nanning	0.053	0.053	0.060	0.048	0.061	0.062	0.060		0.292	0.428	0.419	0.464	0.452	0.463	0.503	0.455	0.468	0.466	0.452	0.452	0.456
Kunming	0.020	0.019	0.042	0.013	0.053	0.061	0.037	0.052		0.285	0.275	0.309	0.285	0.303	0.322	0.278	0.302	0.301	0.295	0.295	0.296
Sanya	0.052	0.051	0.060	0.049	0.061	0.062	0.060	0.061	0.051		0.434	0.443	0.441	0.449	0.481	0.443	0.443	0.464	0.454	0.454	0.437
Haikou	0.054	0.053	0.060	0.049	0.061	0.062	0.060	0.061	0.050	0.061		0.470	0.450	0.461	0.494	0.451	0.458	0.448	0.444	0.444	0.456
Qionghai	0.050	0.048	0.058	0.053	0.062	0.060	0.062	0.062	0.053	0.062	0.062		0.410	0.421	0.458	0.421	0.415	0.449	0.443	0.443	0.416
Wuhan	0.050	0.049	0.058	0.052	0.062	0.061	0.061	0.062	0.051	0.062	0.062	0.060		0.411	0.449	0.411	0.412	0.435	0.435	0.435	0.412
Quzhou	0.050	0.049	0.058	0.053	0.062	0.060	0.062	0.062	0.053	0.062	0.062	0.061	0.060		0.457	0.423	0.422	0.436	0.454	0.454	0.415
Ganzhou	0.054	0.054	0.059	0.056	0.062	0.060	0.062	0.062	0.055	0.062	0.062	0.062	0.062	0.062		0.445	0.452	0.448	0.504	0.504	0.442
Nanchang	0.051	0.050	0.058	0.052	0.062	0.061	0.061	0.062	0.050	0.062	0.062	0.061	0.061	0.061	0.062		0.425	0.438	0.443	0.443	0.420
Rongjiang	0.050	0.049	0.058	0.053	0.062	0.060	0.061	0.062	0.053	0.062	0.062	0.061	0.061	0.061	0.062	0.061		0.438	0.435	0.435	0.416
Heyuan	0.052	0.052	0.059	0.053	0.062	0.061	0.061	0.062	0.053	0.062	0.062	0.062	0.061	0.061	0.062	0.062	0.062		0.462	0.462	0.434
Yangjiang	0.052	0.051	0.060	0.053	0.062	0.059	0.062	0.062	0.052	0.062	0.062	0.062	0.061	0.062	0.062	0.062	0.061	0.062		0.400	0.451
Zhanjiang	0.052	0.051	0.060	0.053	0.062	0.059	0.062	0.062	0.052	0.062	0.062	0.062	0.061	0.062	0.062	0.062	0.061	0.062	0.060		0.451
Guangzhou	0.050	0.049	0.058	0.052	0.062	0.061	0.061	0.062	0.052	0.062	0.062	0.061	0.061	0.061	0.062	0.061	0.061	0.061	0.062	0.062	

Table S4 Nei's genetic distance between 21 geographic populations of red imported fire ants

Colonies	Fuzhou	Meizhou	Longyan	Zhangzhou	Lufeng	Yongzhou	Guilin	Nanning	Kunming	Sanya	Haikou	Qionghai	Wuhan	Quzhou	Ganzhou	Nanchang	Rongjiang	Heyuan	Yangjiang	Zhanjiang	Guangzhou
Fuzhou																					
Meizhou	0.200																				
Longyan	0.525	0.456																			
Zhangzhou	0.481	0.434	0.875																		
Lufeng	1.158	1.058	1.357	0.399																	
Yongzhou	2.011	2.229	1.704	0.584	0.459																
Guilin	0.700	0.871	1.446	0.440	0.983	0.734															
Nanning	1.026	0.888	1.135	0.384	0.084	0.561	0.939														
Kunming	0.640	0.547	0.934	0.342	0.807	1.038	0.542	0.726													
Sanya	0.890	0.720	1.054	0.582	0.260	0.612	0.922	0.296	0.679												
Haikou	1.156	0.973	1.047	0.523	0.232	0.665	0.980	0.170	0.490	0.378											
Qionghai	0.508	0.343	0.497	1.065	0.765	1.899	2.327	0.741	0.867	0.399	0.781										
Wuhan	0.483	0.382	0.390	0.909	0.744	1.438	2.127	0.648	0.608	0.477	0.549	0.077									
Quzhou	0.441	0.414	0.370	0.945	0.877	1.538	2.186	0.749	0.808	0.556	0.684	0.153	0.079								
Ganzhou	0.980	0.974	0.500	1.794	1.977	1.415	2.136	2.034	1.162	1.308	1.439	0.564	0.518	0.567							
Nanchang	0.610	0.536	0.510	0.977	0.743	1.314	1.804	0.696	0.520	0.533	0.567	0.164	0.096	0.187	0.454						
Rongjiang	0.558	0.417	0.481	1.145	1.065	1.717	1.791	0.942	0.851	0.506	0.685	0.112	0.107	0.185	0.534	0.236					
Heyuan	0.829	0.743	0.586	1.215	1.344	1.767	2.228	0.962	0.885	1.141	0.554	0.487	0.378	0.337	0.459	0.408	0.411				
Yangjiang	0.620	0.514	0.769	0.855	0.618	1.948	1.539	0.513	0.622	0.521	0.357	0.299	0.259	0.411	1.249	0.361	0.263	0.583			
Zhanjiang	0.620	0.514	0.769	0.855	0.618	1.948	1.539	0.513	0.622	0.521	0.357	0.299	0.259	0.411	1.249	0.361	0.263	0.583	0.000		
Guangzhou	0.515	0.444	0.457	1.102	0.941	1.727	2.091	0.867	0.869	0.520	0.819	0.133	0.123	0.130	0.486	0.210	0.164	0.421	0.501	0.501	

Table S5 The social form distribution of red imported fire ant population in 21 regions

Regions	Monogyne: Polygyne (Multiple PCR)	Monogyne: Polygyne (The b alleles)
Fuzhou	2:1	2:1
Meizhou	2:1	2:1
Longyan	3:0	3:0
Zhangzhou	0:3	0:3
Lufeng	3:0	3:0
Yongzhou	3:0	3:0
Guilin	3:0	3:0
Nanning	1:2	1:2
Kunming	2:1	3:0
Sanya	0:3	0:3
Haikou	3:0	3:0
Qionghai	3:0	3:0
Wuhan	0:3	0:3
Quzhou	0:3	0:3
Ganzhou	3:0	3:0
Nanchang	0:3	0:3
Rongjiang	1:2	1:2
Heyuan	3:0	3:0
Yangjiang	2:1	3:0
Zhanjiang	3:0	3:0
Guangzhou	1:2	1:2
Total	38:25	40:23

Table S6 Diversity analysis of gut symbiotic bacteria of red imported fire ant from different regions

Sites	sobs	Shannon	Simpson	chao	ace
Fuzhou	858.00±156.63	2.01±1.14	0.41±0.29	1569.70±229.63	1615.67±274.09
Meizhou	900.67±151.58	3.97±2.06	0.66±0.34	1449.99±170.78	1564.98±189.65
Longyan	1168.67±338.74	3.71±1.84	0.67±0.32	1888.96±497.20	2026.78±588.11
Zhangzhou	775.00±21.92	1.95±1.14	0.39±0.24	1310.14±13.34	1389.04±48.88
Lufeng	720.67±33.09	6.25±0.42	0.97±0.01	1127.15±48.55	1214.54±77.07
Yongzhou	773.33±25.59	4.37±0.93	0.88±0.06	1383.71±103.08	1502.13±102.43
Guilin	848.33±25.63	3.85±2.06	0.70±0.27	1374.96±76.99	1438.95±74.61
Nanning	794.00±78.11	4.75±2.66	0.71±0.36	1365.98±308.62	1387.46±329.25
Kunming	722.67±37.63	3.06±0.16	0.79±0.01	1335.54±123.62	1364.75±95.17
Sanya	870.00±16.27	3.84±0.76	0.75±0.13	1504.75±149.95	1566.91±105.84
Haikou	762.00±19.20	5.45±1.53	0.93±0.07	1281.35±157.11	1334.33±150.11
Qionghai	875.00±6.68	3.65±0.48	0.81±0.04	1523.48±135.58	1591.84±94.47
Wuhan	835.67±78.27	3.18±0.93	0.64±0.16	1216.73±127.75	1294.74±122.06
Quzhou	800.67±57.66	1.77±0.60	0.41±0.18	1438.40±138.17	1452.11±132.67
Ganzhou	868.33±104.96	7.08±0.28	0.98±0.00	1220.26±133.55	1222.32±121.90
Nanchang	788.00±76.38	3.85±1.28	0.70±0.23	1243.16±266.35	1271.73±167.89
Rongjiang	781.33±88.19	4.58±2.31	0.72±0.35	1384.26±243.08	1436.24±173.77
Heyuan	739.00	5.21	0.88	1449.00	1573.95
Yangjiang	945.33±48.62	2.97±1.29	0.55±0.27	1548.06±179.65	1580.11±163.71
Zhanjiang	1248.00±384.99	5.26±2.75	0.70±0.31	1599.64±150.89	1594.80±165.77
Guangzhou	783.00±108.89	2.99±1.07	0.64±0.13	1254.41±166.49	1308.45±157.22

Table S7 The latitude of red imported fire ant collection sites and sample information

Populations	Latitude （N）	Number of colonies collected	Number of workers used per colony		
			Microsatellite*	*CO I**	Social form#
Sanya (SY)	18.31	3	20	20	10
Qionghai (QH)	19.14	3	20	20	10
Haikou (HK)	19.92	3	20	20	10
Yangjiang (YJ)	21.88	3	20	20	10
Zhanjiang (ZJ)	22.14	3	20	20	10
Nanning (NN)	22.84	3	20	20	10
Lufeng (LF)	22.95	3	20	20	10
Guangzhou (GZ2)	23.17	3	20	20	10
Zhangzhou (ZZ)	23.61	3	20	20	10
Heyuan (HY)	23.64	3	20	20	10
Meizhou (MZ)	24.33	3	20	20	10
Yongzhou (YZ)	24.71	3	20	20	10
Longyan (LY)	25.07	3	20	20	10
Guilin (GL)	25.1	3	20	20	10
Kunming (KM)	25.14	3	20	20	10
Ganzhou (GZ1)	25.798	3	20	20	10
Rongjiang (RJ)	25.969	3	20	20	10
Fuzhou (FZ)	26.06	3	20	20	10
Nanchang (NC)	28.608	3	20	20	10
Quzhou (QZ)	29.08	3	20	20	10
Wuhan (WH)	30.49	3	20	20	10

* Shared DNA samples. # Shared DNA samples by two identification methods of social form.

Table S8 SSR-PCR primers

Loci	Primers (5'-3')
Sol-6	F: TTTACAGCGAATGGAACACG
	R: FAM-CATTAAGTGCAATTATGCTCGC
Sol-11	F: ACTGGAGCCTCCGAGACC
	R: FAM-CACTCCGGAAGAGTAACTTGC
Sol-20	F: TCGAAACGCTCCCTCTGT
	R: HEX-AGCATGAAAAATCGGGAGC
Sol-42	F: TAMRA-ATGCCGGTTTTTATTGGTAGG
	R: GGAATTTCCGTCGCCATT
Sol-49	F: GTCATCGGTGACCCACAAG
	R: HEX-GTATCTCGACACGAAACTCGG
Sol-55	F: TAMRA-TGCGAATATCCGGTCGAG
	R: CTCGCTTTCTCAGTAAGCCG

F and R represent backward and forward primers, respectively

Table S9 Primers for social form identification (multiplex PCR)

Loci	Primers (5'-3')
26BS	CTCGCCGATTCTAACGAAGGA
16BAS	ATGTATACTTTAAAGCATTCCTAATATTTTGTC
24bS	TGGAGCTGATTATGATGAAGAGAAAATA
25bAS	GCTGTTTTTAATTGCATTTCTTATGCAG

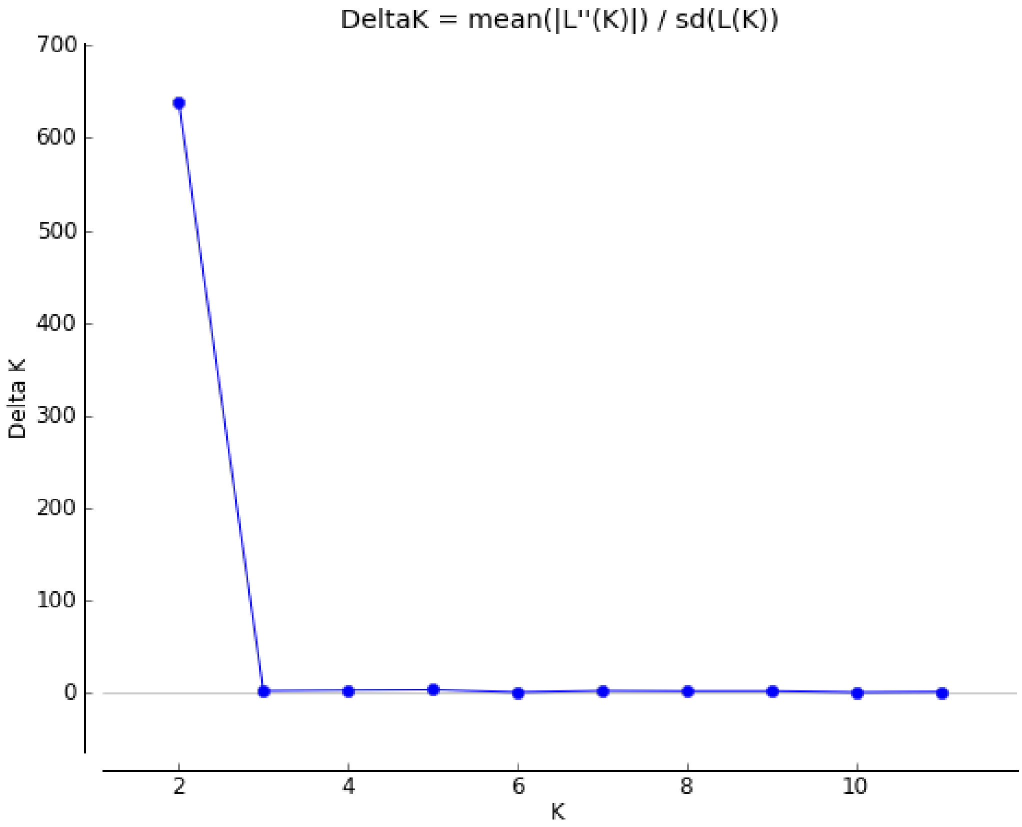

Figure S5 Delta-K values based on 20 runs of K ranging from 1 to 12 using STRUCTURE

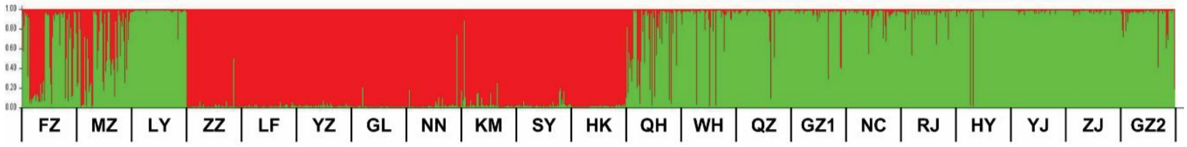

Figure S6 Colony genetic structure of red imported fire ant based on genotypes for K = 2 using the program STRUCTURE. Each individual is represented by a vertical line. The same color stands for individuals that are assigned to the same cluster. Most individuals of FZ, MZ, ZZ, LF, YZ, GL, NN, KM, SY and HK populations were assigned to cluster 1, while LY, QH, WH, QZ, GZ1, NC, RJ,HY, YJ, ZJ and GZ2 populations were assigned to cluster 2.

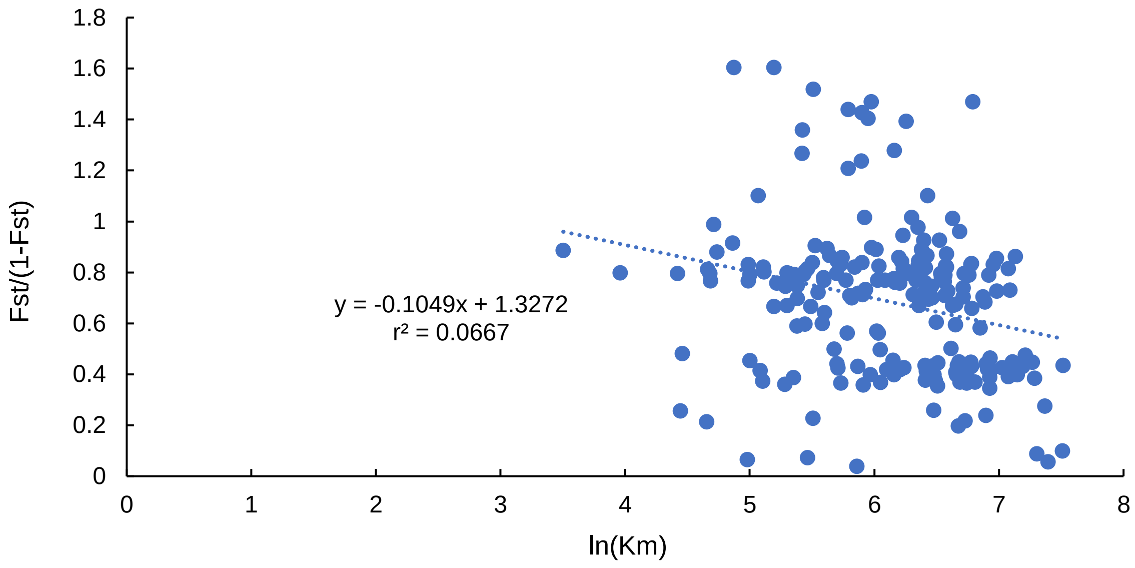

Figure S7 The relationship between genetic differentiation and geographic distance of *Solenopsis invicta* populations

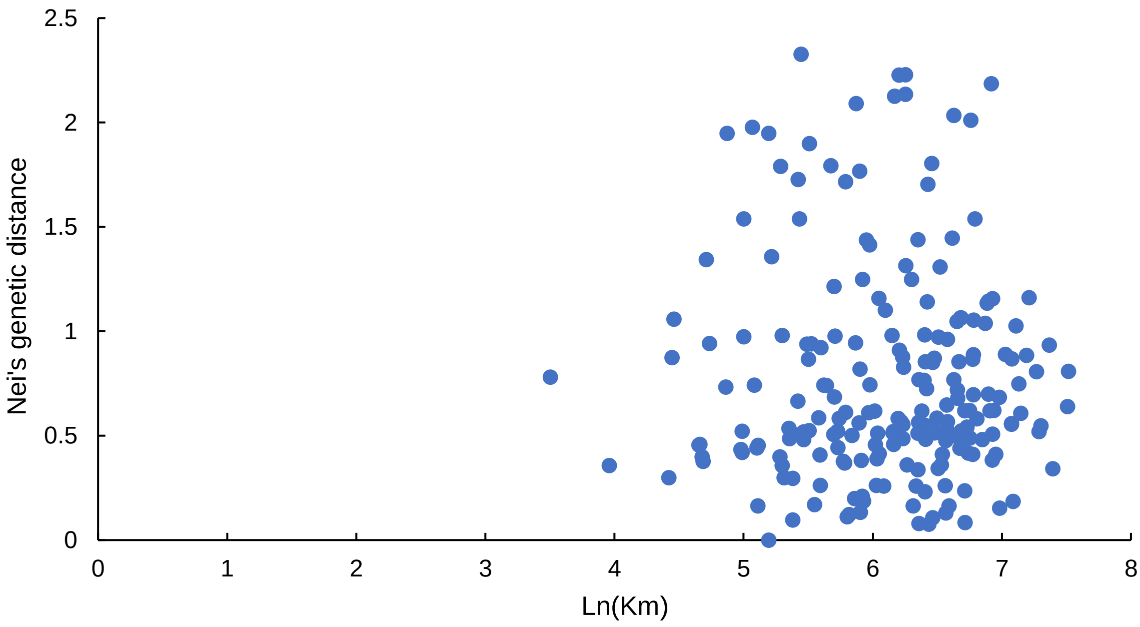

Figure S8 The relationship between Nei's genetic distance and geographic distance of *Solenopsis invicta* populations
